# Computational investigation and machine learning prediction of nanoparticle effects on hemodynamics in patient-specific aortic aneurysms

**DOI:** 10.1371/journal.pone.0350224

**Published:** 2026-10-07

**Authors:** Mohammad Abdul Karim Khan, Kazi Ekramul Hoque, Mohammad Osman Gani, Nayema Islam Nima, Mohammed Abdul Hannan

**Affiliations:** 1 Department of Mathematics, Jahangirnagar University, Dhaka, Bangladesh; 2 Department of Arts and Sciences, Faculty of Engineering, Ahsanullah University of Science and Technology (AUST), Dhaka, Bangladesh; 3 Department of Physical Sciences, Independent University Bangladesh (IUB), Dhaka, Bangladesh; 4 Department of Mechanical Engineering, The University of British Columbia, Vancouver, BC, Canada; Children’s Hospital of Orange County, UNITED STATES OF AMERICA

## Abstract

This study presents a novel computational framework for nanoparticle-assisted drug delivery in abdominal aortic aneurysm (AAA) by characterizing patient-specific hemodynamics under realistic physiological conditions with integrating an artificial neural network (ANN). The model incorporates silver (Ag) nanoparticles within a fully transient, multiphase computational fluid dynamics (CFD) environment, where pulsatile blood flow is imposed using advanced user-defined function. Blood is modeled as a non-Newtonian fluid to capture its shear-dependent rheological behavior, enabling a more accurate representation of vascular flow dynamics. High-resolution patient-specific geometries are developed to investigate four distinct cases, including healthy and AAA arteries with varying geometrical complexities and multiple daughter vessels, both with and without nanoparticle infusion. The analysis focuses on key clinically relevant hemodynamic indicators, including velocity, normal and tangential wall shear stress (WSS). The results indicate that arterial geometry and blood rheology play a dominant role in governing flow behavior, with the most complex vascular configuration exhibiting the strongest hemodynamic response. Among the investigated cases, model 3 shows the highest velocity, decreasing by 52.94% compared to the healthy artery (model 1), while models 2 and 4 exhibit decreases of 82.35% and 54.90%, respectively at left iliac. The introduction of Ag nanoparticles enhances WSS across all models by 34.72%, 47.32%, 51.92%, and 39.67%, respectively, indicating improved flow modulation and more favorable conditions for drug transport and vascular interaction. Flow visualization reveals distinct regions of acceleration, recirculation, and nanoparticle accumulation, which are critical for understanding localized delivery mechanisms. The ANN confirming the robustness, accuracy, and predictive reliability of the developed models. Finally, the proposed framework establishes a clear relationship between arterial morphology, non-Newtonian blood behavior, and nanoparticle-driven hemodynamic modulation, offering valuable insights for optimizing patient-specific, nanoparticle-based therapeutic strategies in cardiovascular disease management.

## Introduction

Abdominal aortic aneurysm (AAA) is a serious and potentially fatal condition characterized by the unusual expansion of the abdominal aorta. Currently, there is no established pharmacological treatment proven to stop or slow its progression effectively. Management often focuses on monitoring and surgical intervention when necessary. This underscores the urgent need for the development of more effective therapeutic strategies capable of delivering drugs directly to the affected regions within the arterial system. In this context, nanoparticle-based drug delivery systems have emerged as a highly promising approach due to their can deliver drugs specifically to target cells, enhance bioavailability, manage the release of drugs and lessen systemic adverse effects. Owing to their tunable physicochemical properties, enhanced permeability, and ability to facilitate targeted delivery, nanoparticles can improve drug bioavailability while minimizing systemic side effects. Consequently, these systems hold significant potential for advancing the treatment of vascular diseases by enabling precise and controlled drug delivery to diseased arterial sites. To identify hemodynamic variations in curved abdominal aortic aneurysms, Algabri et al.[1] used computational fluid dynamics (CFD) to compare stable and pulsatile models. Murakami et al. [2] illustrated that regarding the mean flow velocity in the segment that was modeled, a larger aneurysm led to the formation of a vortex within the aneurysm and a decrease in the flow velocity surrounding the aneurysm. In the idealized angulated neck AAA model, velocity distributions revealed notable distinctions between the two scenarios (stationary and transient) [3]. The simulation results [4,5] indicated that aneurysm angulation generates regions of high and low wall shear stress, along with turbulent flow and recirculation within the sac, potentially contributing to aortic wall weakening. Visual analysis revealed [6,7] that regions of low wall shear stress (WSS) corresponded to high wall pressure (WP). Although velocity and blood pressure influenced both WSS and WP, the central contour consistently indicated potential rupture sites. The risk of AAA rupture was mainly assessed by aneurysm diameter; intervention was typically recommended when it exceeds 5.5 cm or shows rapid expansion [8,9]. ILT is associated with regions of low velocity and TAWSS, and high OSI, ECAP, and RRT values [10–13]. Researchers anticipated [14,15] that advances in nanotechnology, gene editing, precision medicine, and AI will enable engineered nanomedicine to enhance drug targeting, prolong circulation, and achieve controlled release more effectively. According to the experts [16,17], there is a significant danger of rupture and thrombosis when the dilatation exceeds 50% of the normal aortic diameter. AAA is a localized aortic dilation with high rupture mortality (~80–90%). Nanoparticles offer promising targeted therapy to prevent rupture [18,19]. Non-spherical particles exhibit enhanced interactions with the arterial wall, promoting increased collision and adhesion to the target site [20]. The findings showed [21] that aortic aneurysms show significant turbulence during the diastolic phase, and the level of turbulence becomes stronger as the aneurysm grows. The findings exposed that With increasing aneurysm diameter (5–20 mm), OSI rose from 0.3689 to 0.4104 (Ha = 0) and 0.3603 to 0.4532 (Ha = 5), while RRT increased significantly from 4.1455 to 24.2634 and 5.041 to 42.7325, respectively [22]. The results indicated [23] that fusiform aneurysms with higher aspect ratios show lower rupture susceptibility compared to saccular aneurysms with lower aspect ratios. Proximal aneurysms show higher rupture risk than distal ones, emphasizing the role of local hemodynamics [24]. The results suggested [25] that proximal stenosis is more severe than distal stenosis, even when both produce the same lumen reduction. The greatest impact on abnormal arterial blood flow is observed when there are multiple stenoses in a row, as compared to aneurysms, which indicates a more severe form of atherosclerosis [26]. Patients with fast-growing AAAs are thought to have unique immunometabolism profiles and biomechanical signs that can be identified years before traditional size thresholds are reached [27–29]. The study showed [30,31]that the ternary nanofluid exhibited improved heat-transfer features as the couple-stress parameter increases; however, a rise in the Hartmann number leads to enhanced thermal diffusion. It was shown [32] that one side of the AAA is always where the reverse velocity vectors are concentrated. While the flow velocity increases with exercise, the systolic flow patterns become disordered. Although there was no noticeable variation in velocity, an increase in viscosity led to a minor rise in total pressure and wall shear stress [33,34]. Motlagh and Deyhim [35] measured the effects of nanoparticle size and concentration, while Pinto and Campos [36] described flow disruptions at bifurcations associated with illness risk. While Majee and Shit [37] investigated how nanoparticle concentration impacts shear stress and heat transfer, Impiombato et al. [38] used design optimization to avoid grafts, increasing flow outcomes. Targeted drug delivery in vascular diseases is challenged by pulsatile flow and complex arterial geometries. The effects of viscosity, Hartmann number and Weissenberg number on blood flow were investigated using BVP4C method and found that velocity reduced and altered its flow behavior [39,40]. The researcher showed [41,42] that the Williamson and viscosity parameters reduced velocity, while thermal conductivity and radiation enhanced temperature [43].The researcher demonstrated [44] that as temperature increased [45], the Brownian and thermophoresis diffusion coefficients initially rise, but their rate of increase slows down as both parameters were further extended. This study investigates nanoparticle-assisted transport in a patient specific abdominal aortic model with aneurysm, incorporating realistic hemodynamics. Silver (Ag) nanoparticles are introduced to evaluate their effects on WSS, velocity, and pressure, while comparisons are made between healthy and diseased arteries. A transient multiphase CFD approach with UDF-based boundary conditions and non-Newtonian models is employed. The results provide insight into nanoparticle transport and deposition, supporting the potential of Ag nanoparticles for enhanced, site-specific therapy with reduced systemic effects.

This study provides understanding of nanoparticle-assisted blood flow by incorporating nanoparticles into a physiologically realistic abdominal artery model with aneurysm. The inclusion of complex geometries reveals how vascular irregularities and flow disturbances govern nanoparticle transport, deposition, and accumulation. A transient multiphase CFD framework with pulsatile boundary conditions enhances predictive accuracy. Finally, the work strengthens the basis for patient-specific, nanoparticle-based drug delivery strategies in abdominal artery disease.

## Computational methodology

The Navier–Stokes equations underpin the simulation of blood flow in patient-specific abdominal aortic geometries, capturing the transient, incompressible behavior of viscous fluids under pressure and viscous forces. Coupled with the continuity equation, they ensure accurate representation of mass conservation in the flow field.

### Governing equations for CFD simulation

The fundamental governing equations, together with the relevant constitutive relations that describe the fluid behavior, are systematically presented in the following section to establish the mathematical framework of this study.

The incompressible form of continuity equation [46]


$$\begin{array}{c} \nabla \cdot u=\text{0}. \end{array}$$ (1)

The Navier–Stokes equations governing momentum are employed to ensure the conservation of momentum in an unsteady, incompressible fluid flow. The momentum equation [47]


$$\begin{array}{c} \rho \left(\frac{\partial u}{\partial t}\right)+\rho \left(u.\nabla \right)u=\nabla .\left[-pI+K\right]+F. \end{array}$$ (2)

Here *u* stands for velocity, ρ  be  the blood density and its value are 1063 kg/m^3^, p be the pressure and *F* be the body force per unit volume. For an incompressible fluid, the energy equation is


$$\begin{array}{c} \;\rho c_{p}(\frac{\partial T}{\partial t}\;+u\;.\;\nabla T)=k\nabla^{\text{2}}T\;+\;\phi +\;Q \end{array}$$ (3)

Here $c_{p}$ stands for specific heat capacity, T is the temperature, u is the velocity field, k is the thermal conductivity, ϕ represents viscous dissipation, and Q denotes any internal heat generation term. In biomedical blood flow with nanoparticles, viscous dissipation and heat sources are negligible, so the energy equation reduces to:


$$\begin{array}{c} \rho c_{p\;}(\frac{\partial T}{\partial t}+\;u\;.\;\nabla T)\;=\;k\nabla^{\text{2}}T \end{array}$$ (4)

For steady-state conditions, the temporal term vanishes, then


$$\begin{array}{c} \rho c_{p}(\;u\;.\;\nabla T)\;=\;k\nabla^{\text{2}}T \end{array}$$ (5)

The advection–diffusion equation describes nanoparticle transport in blood by combining convection due to flow and diffusion due to Brownian motion, where advection represents flow-driven transport, diffusion represents concentration spreading, and the time term represents local concentration change. The advection – diffusion equation is [48]


$$\begin{array}{c} \partial C/\partial t+u\;.\nabla C=D\nabla^{\text{2}}C. \end{array}$$ (6)

$$\begin{array}{c} \text{Where},\;D=\frac{K_{B}T}{\text{6}\pi \mu r}. \end{array}$$ (7)

Here *C* stands the concentration, *D* represents diffusion coefficient of nanoparticle with radius *r*, *K*_*B*_ is the Boltzmann constant, μ is the viscosity and *T* is the temperature.

The Brinkman model is applied for moderate nanoparticle concentrations to describe flow through a medium with increased effective viscosity [49]


$$\begin{array}{c} \mu_{nf}=\frac{\mu_{blood}}{{\left(\text{1}-\varphi \right)}^{\text{2}.\text{5}}}. \end{array}$$ (8)

Where $\mu_{nf\;}$= viscosity of nanofluid (blood + nanoparticles), $\mu_{blood}$= viscosity of blood, φ = nanoparticle volume fraction. In nanofluid (silver + blood), an effective thermal conductivity is as follows [49]


$$\begin{array}{c} k_{nf}=k_{f}\left[\frac{k_{p}+{\text{2}k}_{f}-\text{2}\varphi \left(k_{f}-k_{p}\right)}{k_{p}+{\text{2}k}_{f}+\varphi \left(k_{f}-k_{p}\right)}\right]. \end{array}$$ (9)

Where $k_{nf}$= Nanofluid thermal conductivity, $k_{f}$= Blood conductivity, $\;k_{p}$= Nanoparticles conductivity, φ = Nanoparticles volume fraction. For nanoparticles in blood, the effect heat is as follows [49]


$$\begin{array}{c} {\left(\rho c_{p}\right)}_{nf\;}=(\text{1}-\varphi )({\rho c_{p})}_{f}+\varphi (\rho {c_{p})}_{p}. \end{array}$$ (10)

Where ${\left(\rho c_{p}\right)}_{nf\;}$= Effective heat capacity of nanofluid, $({\rho c_{p})}_{f}$ = Heat capacity of blood fluid, $(\rho {c_{p})}_{p}$ = Heat capacity of nanoparticles. The Carreau–Yasuda model is employed to represent the shear-thinning behavior of whole blood, as it reliably captures viscosity variations over a wide range of shear rates [50].


$$\begin{array}{c} \;\mu \dot{\left(\gamma )=\;\mu_{\infty }\;+{(\mu }_{\text{0}\;}-\mu_{\infty }\right)}{\left[\text{1}+{\left(\lambda \dot{\gamma }\right)}^{a}\right]}^{\frac{n-\text{1}}{a}} \end{array}$$ (11)

In this context, $\mu \dot{\left(\gamma \right)}$denotes the shear-rate-dependent viscosity, where$\dot{\;\gamma }$ is the shear rate. Here, $\mu_{\text{0}\;}$ represents the zero-shear viscosity, while${\;\mu }_{\infty }$ corresponds to the infinite-shear viscosity. The parameter λ defines the relaxation time, n is the power-law index, and a is the transition parameter governing the smoothness of the viscosity variation between limiting states.

### Computational models

The computational geometry used in this study is presented in Fig 1. The patient-specific AAA artery models were reconstructed using data obtained from an open-source repository [51]. The geometries, Fig 1, include both healthy and pathological configurations associated with AAA. Specifically, four cases are considered: Fig 1(a) represents a healthy abdominal aorta, while Fig 1[(b) – (d)] correspond to diseased arteries with different aneurysm diameters of 35 mm, 37 mm, and 39.37 mm respectively.

**Fig 1:**
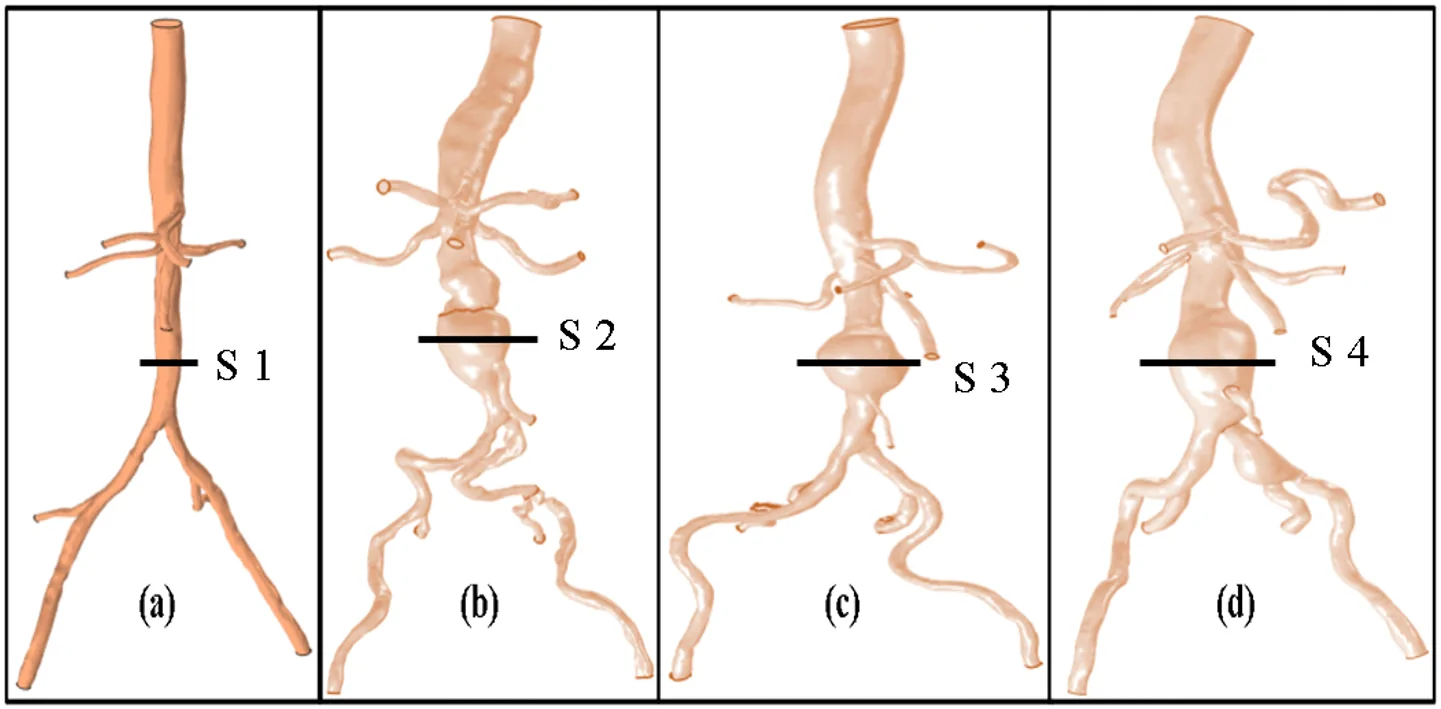
Computational models: (a) Healthy model (Model 1); (b – d) diseased models (Model 2 – Model 4) and S1- S4 present the cross sections in the diseases area.

Fig 2 represents the different parts of patient-based aortic model. All models are analyzed under non-Newtonian blood flow conditions using the Carreau–Yasuda viscosity model, with the additional inclusion of nanoparticles to account for enhanced transport characteristics. Table 1 summarizes the general properties of Model 3, whereas the geometric dimensions of the inlet and outlet sections for all considered cases are provided in Table 2.

**Table 1:** Baseline parameters of Model-3.

Species	Human
Anatomy	Abdominal Aorta
Disease	Abdominal Aortic Aneurysm
Age	48
sex	Male
Height(m)	1.73
Weight(kg)	81.2
P sys SP cuff	140
P sys DP cuff	80
Heart Rate (beats/min)	51
Image Modality	CT
Image Type	VTI
Image Source	MARQ
Image Manufacturer	SIEMENS

**Table 2:** Brief description of the different AAA models.

Description	Model-1 (mm)	Model-2 (mm)	Model-3 (mm)	Model-4 (mm)
**Aorta**	16.77	25.2	25	27.90
**Celiac splenic**	3.69	6.22	4.7	8.7
**Celiac hepatic**	5	8.1	5.15	5.87
**SMA**	4.4	8.31	5.87	5.63
**Renal right**	3.69	6.18	4.72	3.47
**Renal left**	4.4	5.74	4	5.46
**IMA**	00	5.47	1.2	3.16
**Right internal iliac**	4.4	5.33	5.13	7.36
**Right iliac**	7.75	6.57	9.39	8.26
**Left internal iliac**	5	3.37	5.55	7.17
**Left iliac**	8.1	8.05	7.84	11.12
**Aneurysm**	00	35	37	39.37

**Fig 2:**
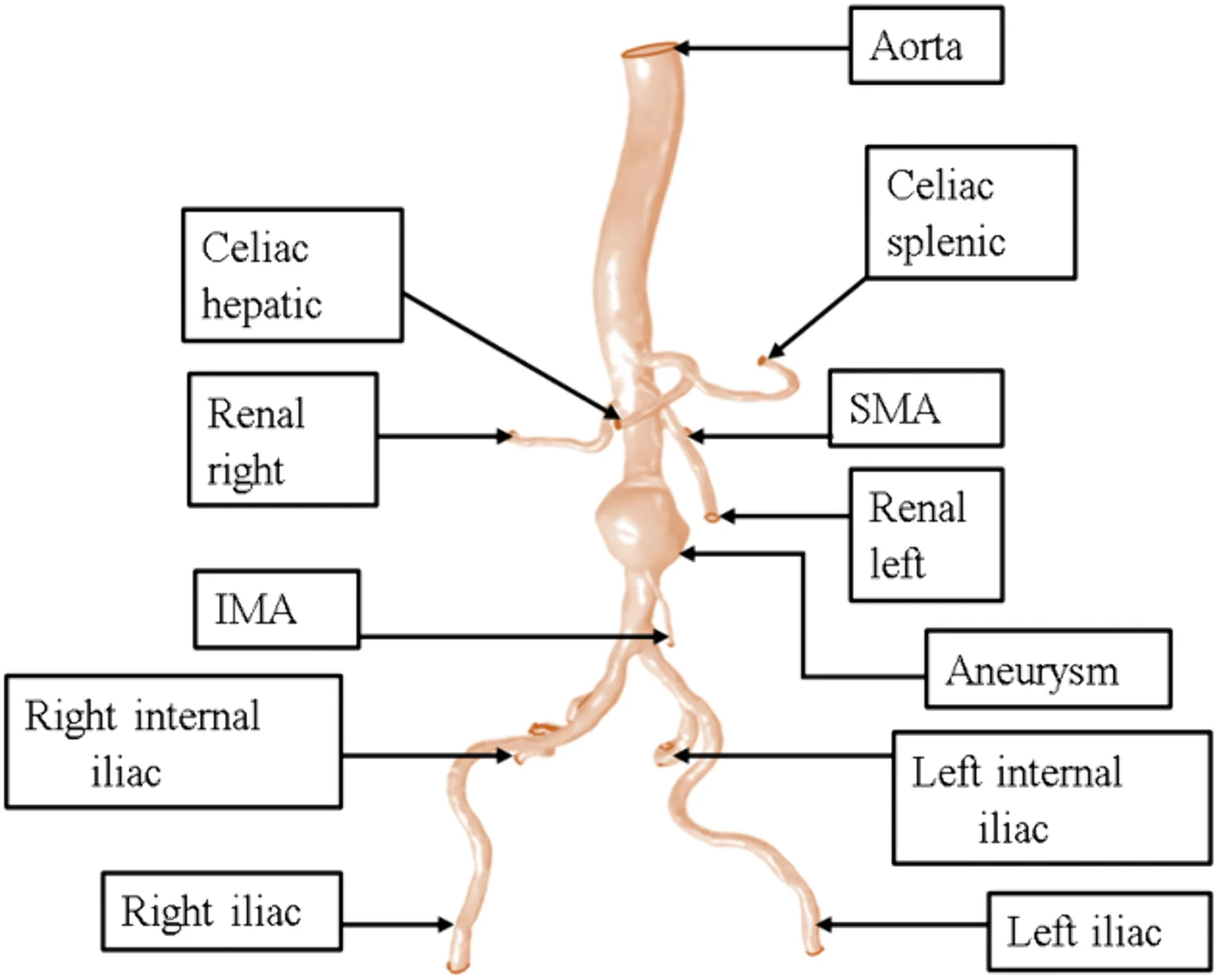
Display the Model 3: diseased artery with a 37 mm aneurysm. [SMA = superior mesenteric artery, IMA = inferior mesenteric artery].

### Boundary conditions for the computational domain

This study considers blood flow under the assumption of laminar, incompressible motion for the non-Newtonian case. The Carreau–Yasuda model is adopted due to its well-established capability to capture the shear-thinning behavior of blood over a wide range of shear rates, as demonstrated in previous studies by [52,53]. Its combination of predictive accuracy, numerical stability, and computational efficiency makes it particularly suitable for simulating complex arterial hemodynamics, including aneurysmal and pulsatile flow conditions. The simulations are carried out in a transient (time-dependent) framework to replicate physiological cardiac cycles.

A clinical study of abdominal artery hemodynamics reported an average blood flow velocity of 0.1798 ± 0.0566 m/s [54] based on digital tracing angiography and nanoparticles size is 40 nm and concentration is 1% volume fraction of silver. In the present simulation, these clinical findings are incorporated, and an inlet velocity of 0.16 m/s and outlet constant pressure is prescribed to represent physiological conditions. The aorta is defined as the inlet boundary, while all branching vessels are assigned as outlets, as summarized in Table 2.

The user defined function (UDF) explores the time-dependent velocity profiles at the point of entrance and the corresponding outlet conditions, as depicted in Fig 3. The velocity gradient’s function is expressed as follows [55]:


$$\begin{array}{c} f(t)=\left\{ \begin{array}{c} (\text{1}.\text{7}\text{5}-\alpha )\text{ sin}(\pi t)\;,\;\text{0}\leq t\leq \text{0}.\text{2}\text{5}\\ \text{1}-\alpha \text{cos}\left(\text{2}\pi (t-\text{0}.\text{5})\right),\;\text{0}.\text{2}\text{5}\leq t\leq \text{1}.\text{5}. \end{array}\right. \end{array}$$

**Fig 3:**
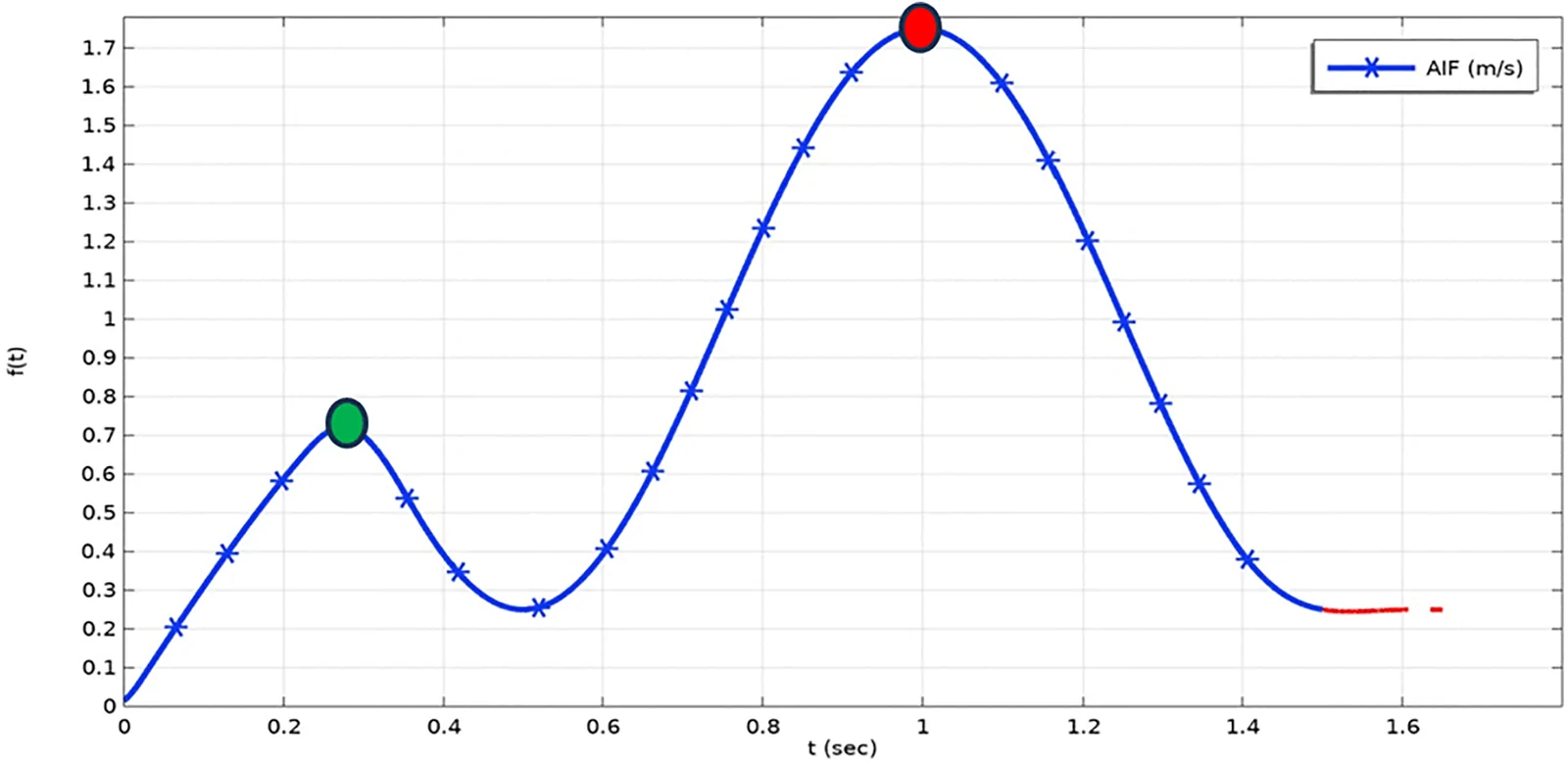
UDF-defined inlet velocity profile for systolic and diastolic phases [55].

Here f(t) be the velocity gradient function and $\alpha =\frac{\text{1}}{\text{3}}$. A pulsatile inlet velocity profile, representative of the human heartbeat [56,57], is prescribed using a UDF, as illustrated in Fig 3 [55]. At all outlet boundaries, a constant pressure condition is imposed. The arterial wall is treated as rigid with a no-slip boundary condition, ensuring zero fluid velocity at the wall interface. For the investigation of nanoparticle-based drug delivery, the Eulerian multiphase formulation is employed.

In this framework, blood is modeled as the primary continuous phase, while silver (Ag) nanoparticles are considered as the dispersed secondary phase. This approach, commonly referred to as Multiphase Computational Fluid Dynamics (MCFD), enables a detailed representation of particle–blood interactions within the arterial domain. All thermophysical properties of blood and nanoparticles are adopted from established by following the source [58], and the corresponding values are summarized in Table 3 and Table 4.

**Table 3:** Material property of the fluid.

Material property name	Silver	Blood
**Density [kg/m** ^**3**^ **]**	10500	1063
Viscosity [Pa*s]	0	0.0035
Thermal conductivity [W/(m*K)	429	0.5
**Specific heat [j/kgK]**	235	3594

**Table 4:** Volume Fraction and properties.

Property	Blood	1% Ag–blood nanofluid
**Ag volume fraction**	—	0.01
**Density, ρ (kg/m³)**	1063	1157.37
**Zero-shear viscosity, μ**_**0**_ **(Pa·s)**	0.0560	0.05742
**Infinite-shear viscosity, μ**_**∞**_ **(Pa·s)**	0.00350	0.003589
**Relaxation time, λ (s)**	3.313	3.313
**Power-law index**	0.3568	0.3568
**Yasuda parameter**	0.64	0.64
**Silver density, ρ (kg/m³)**	—	10,500

The governing equations are solved numerically based on the finite element method (FEM). The fluid domain is analyzed using a laminar flow formulation. A fully coupled solver strategy is implemented to ensure robust coupling between pressure and velocity fields while maintaining second-order accuracy. The iterative convergence criteria are set to a residual tolerance of 1.0 × 10^−6^ for both momentum and continuity equations.

### Grid generation and mesh sensitivity assessment

The FEM is utilized for spatial discretization due to its robustness, accuracy, and effectiveness in handling complex geometries, particularly the diseased patient specific artery. The computational domain is discretized using an unstructured tetrahedral mesh, which is further refined to improve numerical resolution. A 10-node quadratic tetrahedral element is adopted to represent the three-dimensional domain in Fig 4, allowing higher-order interpolation and more precise evaluation of velocity, pressure, and other key hemodynamic variables within patient-specific artery. To ensure the reliability of the numerical solution, a mesh independence study is performed. Several mesh densities are examined, and it is observed in Fig 5 that the results obtained with 285509 elements remain essentially unchanged when compared with finer meshes (Table 5). This indicates that further refinement does not significantly influence the computed pressure and velocity fields. Therefore, the selected mesh is considered sufficiently refined while maintaining computational efficiency. The resulting nonlinear system of equations is solved iteratively using Newton’s method. Convergence is achieved when the difference between successive iterations satisfies the criterion [59].


$$\begin{array}{c} (\gamma^{n+\text{1}}-\gamma^{n})\leq {\text{1}\text{0}}^{-\text{6}} \end{array}$$

**Table 5:** Grids information.

Mesh type	Time	Number of grids	velocity (m/s)	Change (%)	Pressure (Pa)	Change (%)
**Coarser**	35 min 09s	14712	1.1	2.73%	1956	19.38%
**Coarse**	48 min 33s	29037	1.07	7.01%	1577	10.785
**normal**	57 min 22s	85567	0.995	0.30%	1407	2.70%
**Fine**	1 hour 08 min 17s	285509	0.992	0.0%	1369	0.0%
**Finer**	1 hour 42 min 32s	465574	0.992	--	1369	--

**Fig 4:**
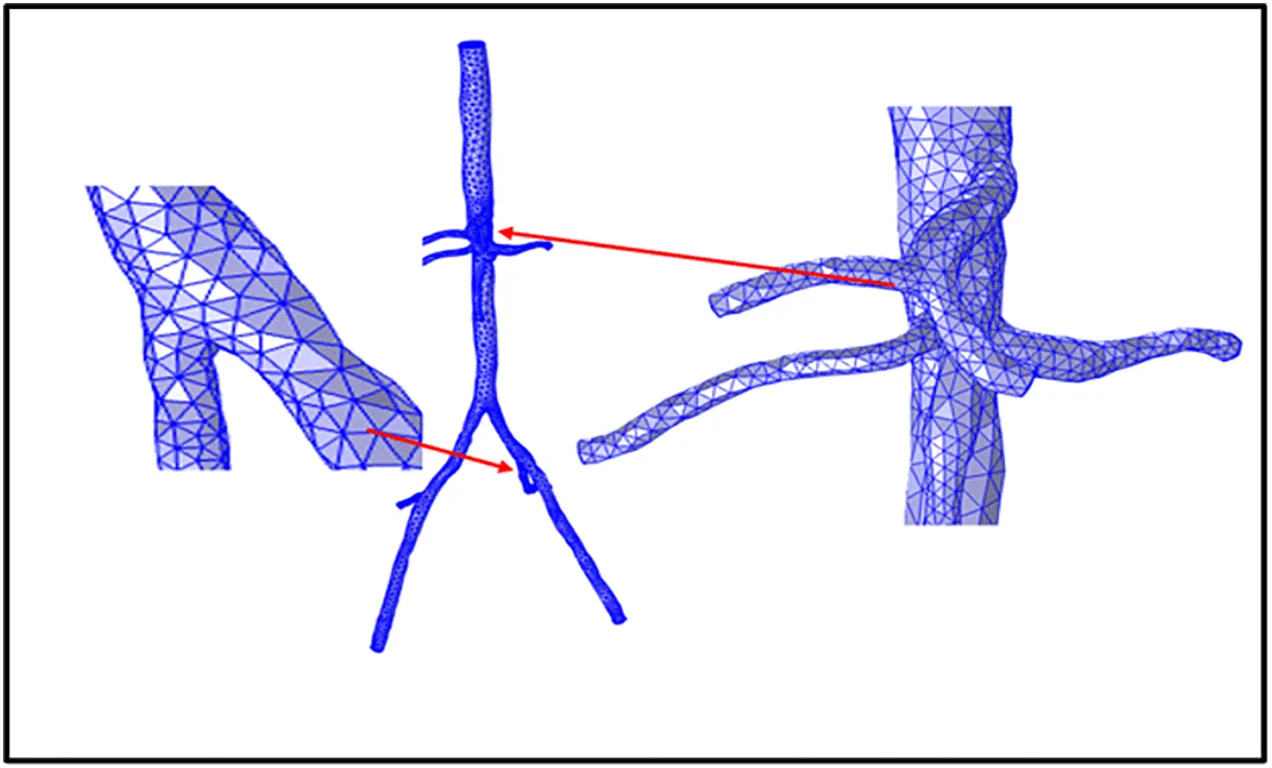
Computational mesh with a zoomed view of local refinement.

**Fig 5:**
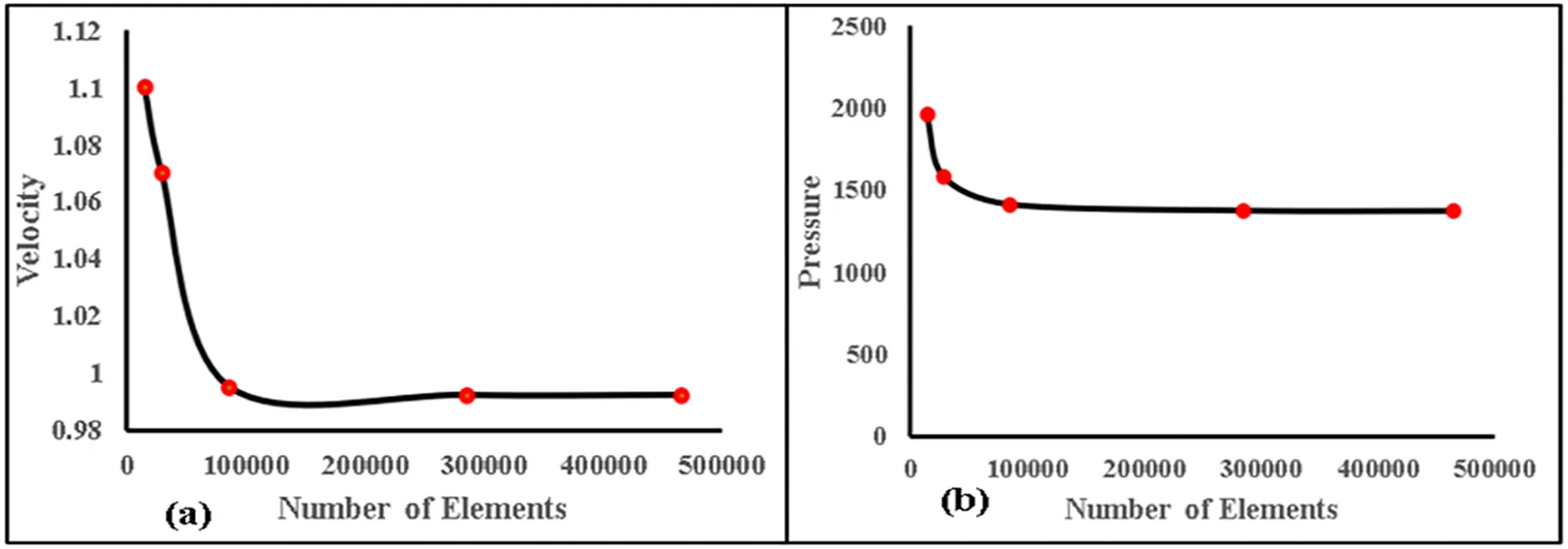
Mesh independence: (a) velocity, (b) pressure vs. element number.

Here, $\gamma^{n}$ denotes the solution at the n^th^ iteration. This tolerance ensures that the solution has stabilized with negligible variation between iterations. Such a convergence threshold is commonly adopted in computational fluid dynamics, providing an appropriate balance between numerical accuracy and computational cost. Finally, the chosen discretization and mesh configuration are adequate for accurately capturing the hemodynamic behavior in the present CHD simulations.

### Numerical validation with existence literature

To establish the credibility of the proposed CHD model, the numerical predictions are benchmarked against previously reported results. The validation process emphasizes the velocity field, given its importance as a key hemodynamic marker for assessing stenosis severity and flow behavior. A comparative analysis is performed with the study of Rahma and Abdelhamid [60] and Hussain et al. [61], which presents detailed simulations of blood flow in a coupled stenotic–aneurysmal arterial geometry. Fig 6[(a)–(d)] shows iso-velocity (streamline) contours for flow pattern analysis. They are evaluated at t = 1s, the greatest flow rate. Flow recirculation is strongly correlated with blood flow velocity. When velocity rises, flow circulation is closely related to the aneurysm’s curvature and complicated shape. Figs 6(c) and 6(d)show two flow regimes. The bulge had recirculating flow (vortex formation), whereas the vessel entrance and exit had laminar flow. High flow velocity (0.888 m/s) aids aneurysm zone recirculation. The rear aneurysm split causes flow recirculation. The velocity profiles and streamlines match Rahma and Abdelhamid [60], establishing confidence in the numerical simulations. The velocity magnitude reduces dramatically near the aneurysm. In Fig 6, estimated velocity magnitudes are compared to the prior research. The velocity magnitudes calculated range from 0 to 1.4, which matches the literature [60]. For consistency, closely matched geometric configurations and computational settings are adopted to ensure a meaningful comparison. The assessment is conducted under pulsatile flow conditions at multiple time instances (t = 2s, 4s, 6s, and 7s), considering velocity magnitude, pressure distribution, and WSS patterns. The Newtonian-based velocity contours are depicted in Fig 6, while Fig 7 [62] illustrates the temporal development of flow structures, providing insight into the dynamic characteristics of the solution and supporting model verification. Quantitative evaluation is carried out along two axial sections positioned at x = 0.2m and x = 1.9m. The computed maximum deviations in velocity are found to be 1.04%, 5.5%, 9.5%, and 9.6% at t = 0.2s, 0.4s, 0.6s, and 0.7s respectively, whereas the corresponding minimum deviations are 1%, 5.2%, 3%, and 1.8% at t = 2s, 4s, 6s, and 7s. These variations remain within acceptable numerical tolerance limits for complex hemodynamic simulations.

**Fig 6:**
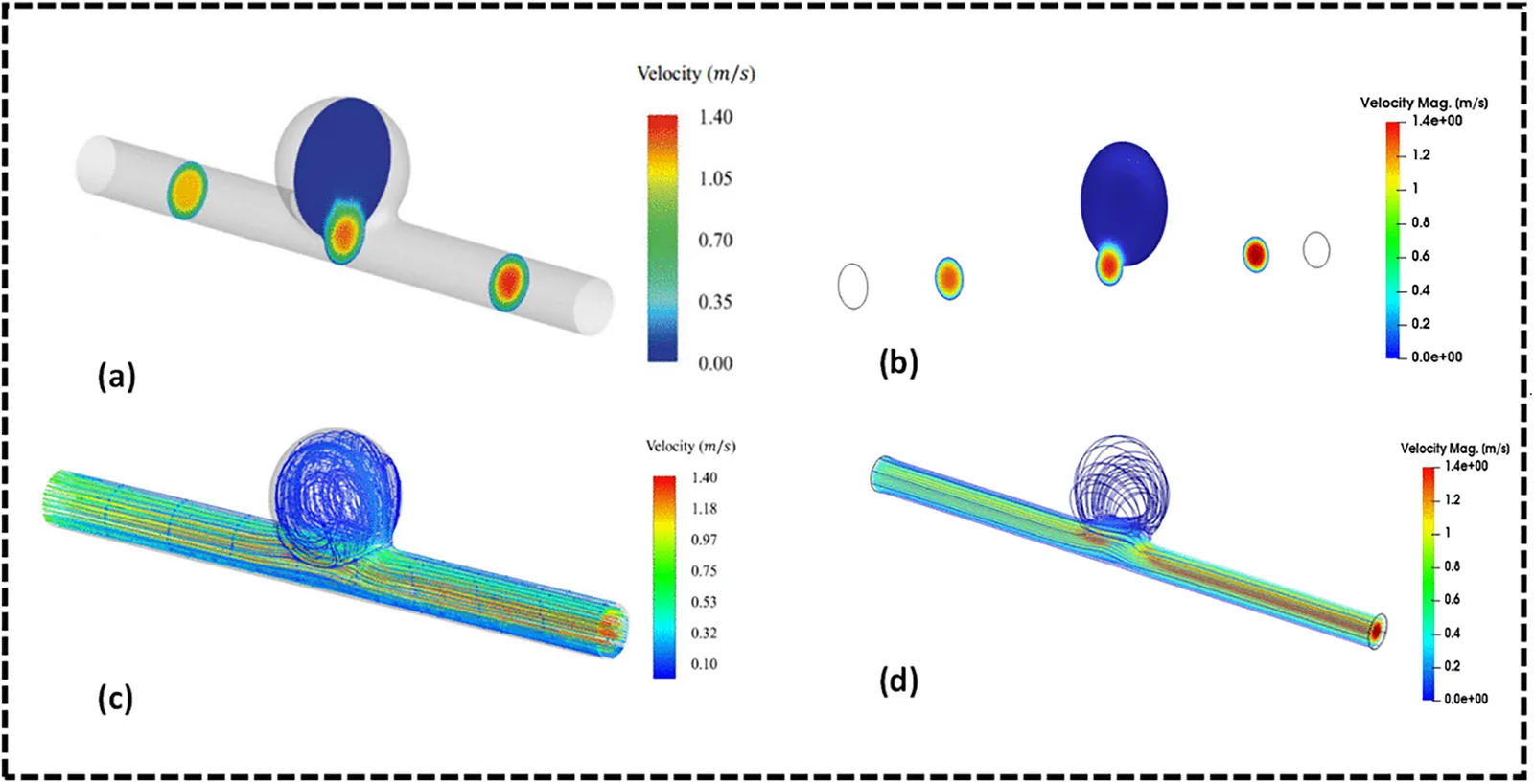
Velocity contour validation against published results [60].

**Fig 7:**
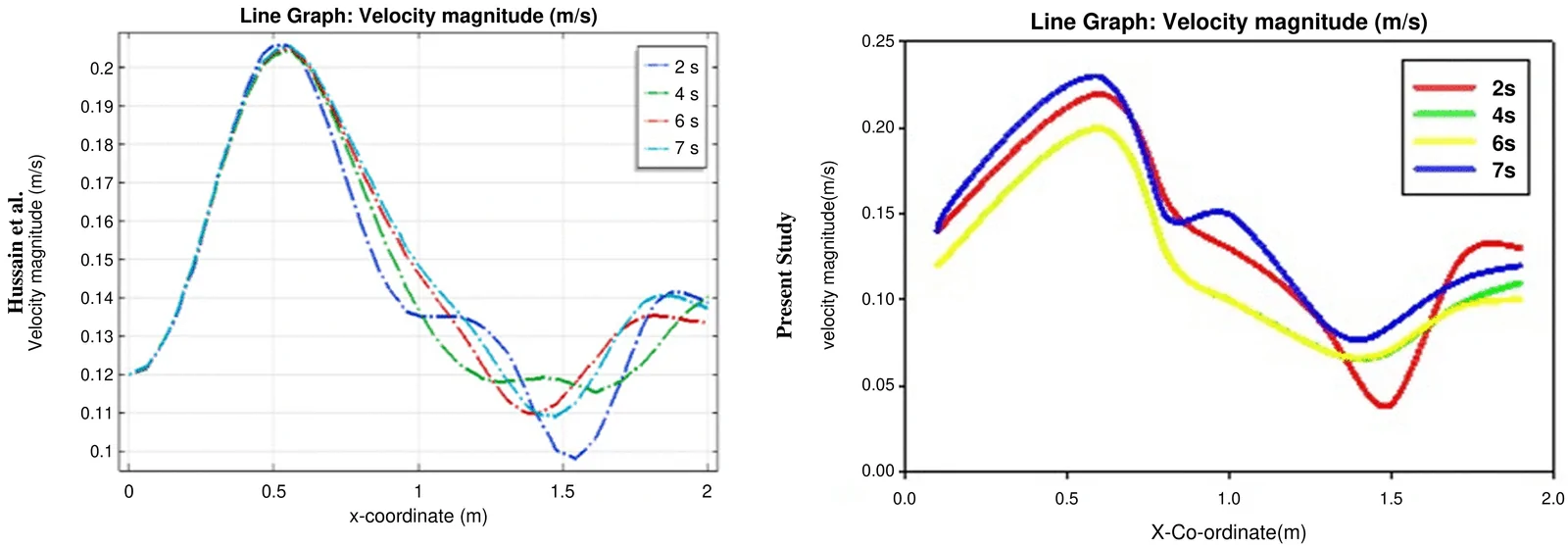
Validation of present results against published data [61].

The minor discrepancies observed can be attributed to subtle differences in geometric reconstruction and localized surface irregularities. Nevertheless, the overall agreement between the present results and those of Hussain et al. [61] demonstrates the stability and predictive capability of the computational approach. This validation confirms that the developed model provides a reliable framework for simulating CHD-related blood flow phenomena.

## Results and discussion

This study investigates key hemodynamic parameters associated with varying aneurysm severities in a patient-specific abdominal artery to assess their influence on aneurysm growth and rupture risk. By analyzing flow behavior within and around the aneurysmal region, it evaluates the mechanical factors governing stability and progression. The findings establish correlations between hemodynamic conditions and aneurysm severity, enhancing the understanding of their combined role in aneurysm development.

### Velocity performances in different healthy and diseased AAA models

Four patient-specific AAA geometries are categorized according to aneurysm sac diameter and subsequently analyzed using CFD to assess the associated flow characteristics and hemodynamic alterations. The aneurysm sac diameter, together with the key geometric features of each case, is summarized in Table 2. Figs 8(1) and 8 [2a-2d] show that all four cases exhibited distinct aneurysmal morphologies, Model 4 was unique in presenting a concomitant iliac artery aneurysm in addition to the abdominal aortic aneurysm, as illustrated in Fig 1(d). The blood flow patterns throughout the cardiac cycle for all four models are depicted in Figs 8(1) and 8 [2a-2d], while the corresponding outlet velocity values are listed in Table 3. A comparative analysis indicates that Model 3 exhibited the highest peak velocity, whereas Model 1 demonstrated the lowest velocity among the investigated cases.

**Fig 8:**
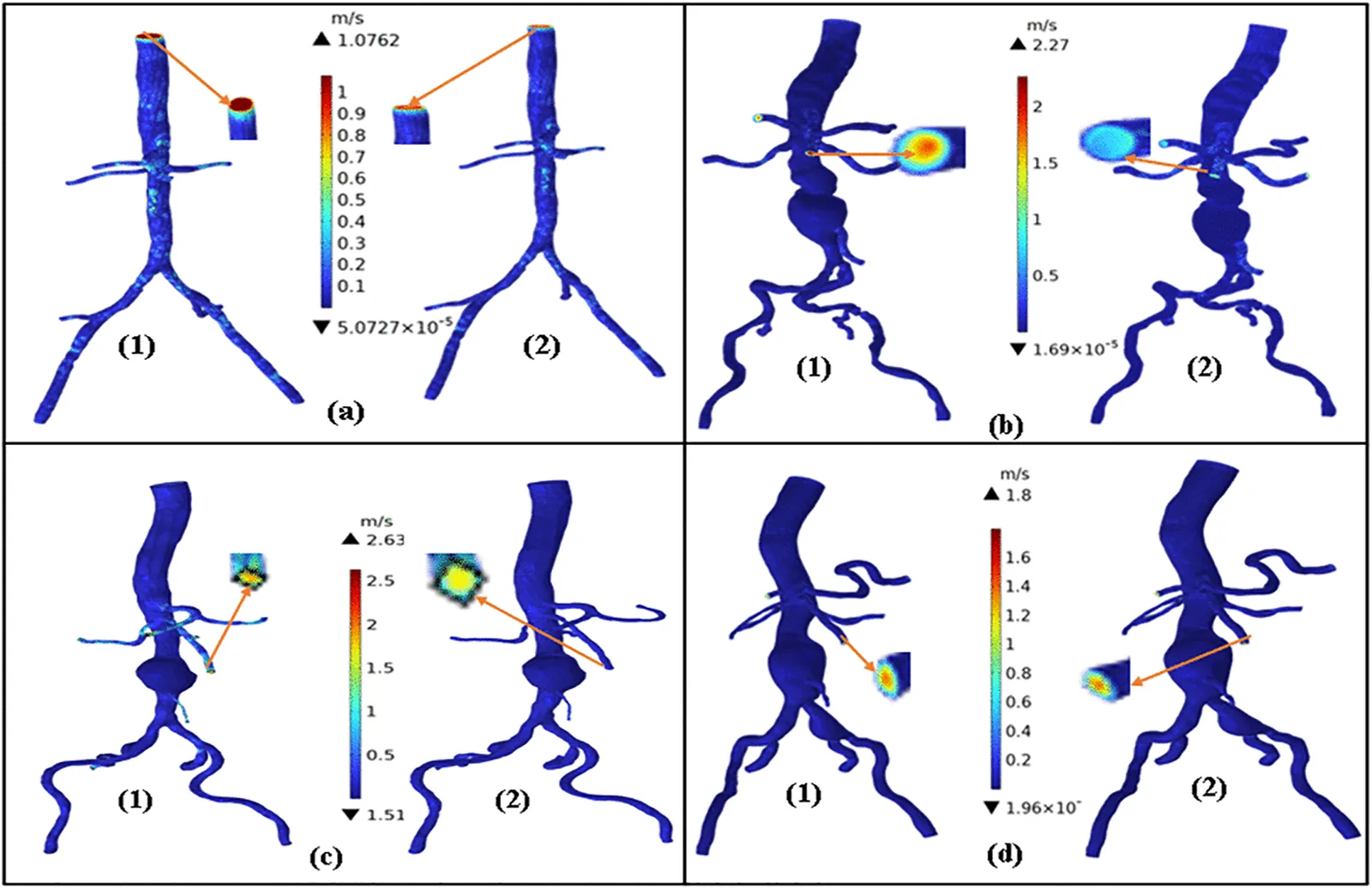
Volumetric velocity contour of different models: Without nanoparticles (a) 1, (b) 1, (c) 1, (d) 1; with nanoparticles (a) 2, (b) 2, (c) 2, (d) 2.

These variations highlight the significant influence of aneurysm geometry on the local hemodynamic environment. In Model 1, representing the healthy aortic configuration, the maximum outlet velocity occurred at the celiac hepatic artery, reaching 1.04 m/s, followed by the superior mesenteric artery (SMA) at 0.94 m/s. For Model 2, which included an aneurysmal dilation, the highest velocity is recorded at the inferior mesenteric artery (IMA), with a peak value of 1.99 m/s, while the right renal artery exhibits the second-highest velocity at 1.13 m/s. In Model 3, the peak velocity shifted to the right renal artery, attaining 2.15 m/s, followed closely by the celiac hepatic artery at 2.03 m/s. Similarly, in Model 4, the IMA experienced the highest velocity of 1.72 m/s, whereas the right renal artery registered the second-highest value of 1.46 m/s. A comparison among the four cases clearly demonstrates that aneurysmal deformation substantially alters the flow distribution within the abdominal aorta and its branches. Relative to the healthy model, the maximum velocity increased by 47.74% in Model 2, 51.63% in Model 3, and 39.53% in Model 4, indicating pronounced flow acceleration in the presence of aneurysmal enlargement. Further examination of branch-specific flow asymmetry reveals notable differences between paired vessels. In Model 1, the celiac hepatic artery exhibits a velocity 36.54% greater than that of the celiac splenic artery. Likewise, the right renal artery exceeded the left renal artery by 40.22%, the left internal iliac artery surpassed the right by 41.18%, and the left common iliac artery showed a 17.65% higher velocity than the right counterpart. For Model 2, the celiac hepatic artery velocity is 65.88% higher than that of the celiac splenic artery. The right renal artery demonstrated a 38.94% increase over the left renal artery, while the left internal iliac artery exceeded the right by 4.76%. In contrast, the velocities in the left and right common iliac arteries are essentially identical. In Model 3, the celiac hepatic artery maintained a 22.66% higher velocity than the celiac splenic artery. The right renal artery exceeded the left by 9.77%, and the left internal iliac artery is 10.78% higher than the right. However, the right common iliac artery displayed a substantially greater velocity, exceeding the left by 36.84%. For Model 4, the celiac hepatic artery exhibits the greatest asymmetry, with a velocity 78.32% higher than that of the celiac splenic artery. The right renal artery is 6.85% faster than the left, the left internal iliac artery exceeded the right by 18.25%, and the left common iliac artery demonstrated a 42.86% higher velocity than the right. These findings underscore the strong influence of aneurysm geometry on regional flow redistribution, leading to elevated velocities and pronounced asymmetry across the branching arterial network. Such hemodynamic alterations may have important implications for aneurysm progression, wall stress development, and the assessment of rupture risk. Following the introduction of nanoparticles, a general reduction in velocity is observed throughout the arterial branches in all investigated models, as summarized in Table 6. This overall decrease can be attributed to the modified rheological characteristics of the nanofluid, which alter momentum transport and redistribute the flow within the vascular network. Nevertheless, an interesting exception is identified in Model 3, where the inferior mesenteric artery (IMA) and the right internal iliac artery exhibit a slight increase in velocity. This localized enhancement is likely associated with improved flow organization, reduced recirculation intensity, and more effective kinetic energy transfer within these branches. Such behavior suggests that nanoparticle suspension can influence regional hemodynamics in a non-uniform manner, depending strongly on the underlying vascular geometry and local flow structures.

**Table 6:** Variation of hemodynamic parameters.

Description	Model 1		Model 2		Model 3		Model 4	
	Velocity (m/s)	Velocity with Nanoparticles (m/s)	Velocity (m/s)	Velocity with Nanoparticles (m/s)	Velocity (m/s)	Velocity with Nanoparticles (m/s)	Velocity (m/s)	Velocity with Nanoparticles (m/s)
**Celiac splenic**	0.66	0.55	0.29	0.28	1.57	1.5	0.31	0.29
**Celiac hepatic**	1.04	1.02	0.85	0.85	2.03	1.90	1.43	1.43
**SMA**	0.94	0.90	1.10	1.09	1.97	1.97	1.33	1.30
**Renal right**	0.92	0.90	1.13	1.13	2.15	2.09	1.46	1.46
**Renal left**	0.55	0.54	0.69	0.68	1.94	1.90	1.36	1.30
**IMA**	00	00	1.99	1.89	1.20	1.23	1.72	1.65
**Right internal iliac**	0.20	0.17	0.40	0.39	1.67	1.68	1.26	1.20
**Right iliac**	0.42	0.41	0.09	0.07	0.38	0.37	0.20	0.18
**Left internal iliac**	0.34	0.29	0.42	0.41	1.49	1.47	1.03	1.00
**Left iliac**	0.51	0.48	0.09	0.08	0.24	0.23	0.35	0.29

Cross-sectional velocity contours provide important insight into the local hemodynamic characteristics of the abdominal aorta under different pathological conditions. Figs 9a-9d present the velocity distributions at representative cross-sections (Fig 1) for all investigated models, illustrating the combined effects of aneurysmal geometry and nanoparticle suspension. In the healthy artery (Fig 9a), the velocity profile exhibits the classical parabolic distribution associated with fully developed laminar flow. The maximum velocity reaches 0.41 m/s for the base fluid. After nanoparticle incorporation, the peak velocity decreases to 0.37 m/s, representing a reduction of approximately 9.76%. This decrease is attributed to the increased effective viscosity of the nanofluid, which suppresses axial acceleration and moderates the overall flow intensity. For Model 2 (Fig 9b), the presence of an aneurysmal dilation produces a distinctly left-skewed velocity profile within the enlarged region. Although the aneurysm alters the flow structure, the peak velocity remains nearly unchanged, indicating that the dilatation primarily redistributes momentum rather than substantially accelerating the flow. When nanoparticles are introduced into the aneurysmal model (Fig 9c), the peak velocity increases by approximately 1.89% relative to the corresponding base-fluid case. This enhancement suggests improved momentum transport within the aneurysm sac, likely resulting from reduced flow separation, weakened recirculation, and more efficient energy transfer.

**Fig 9:**
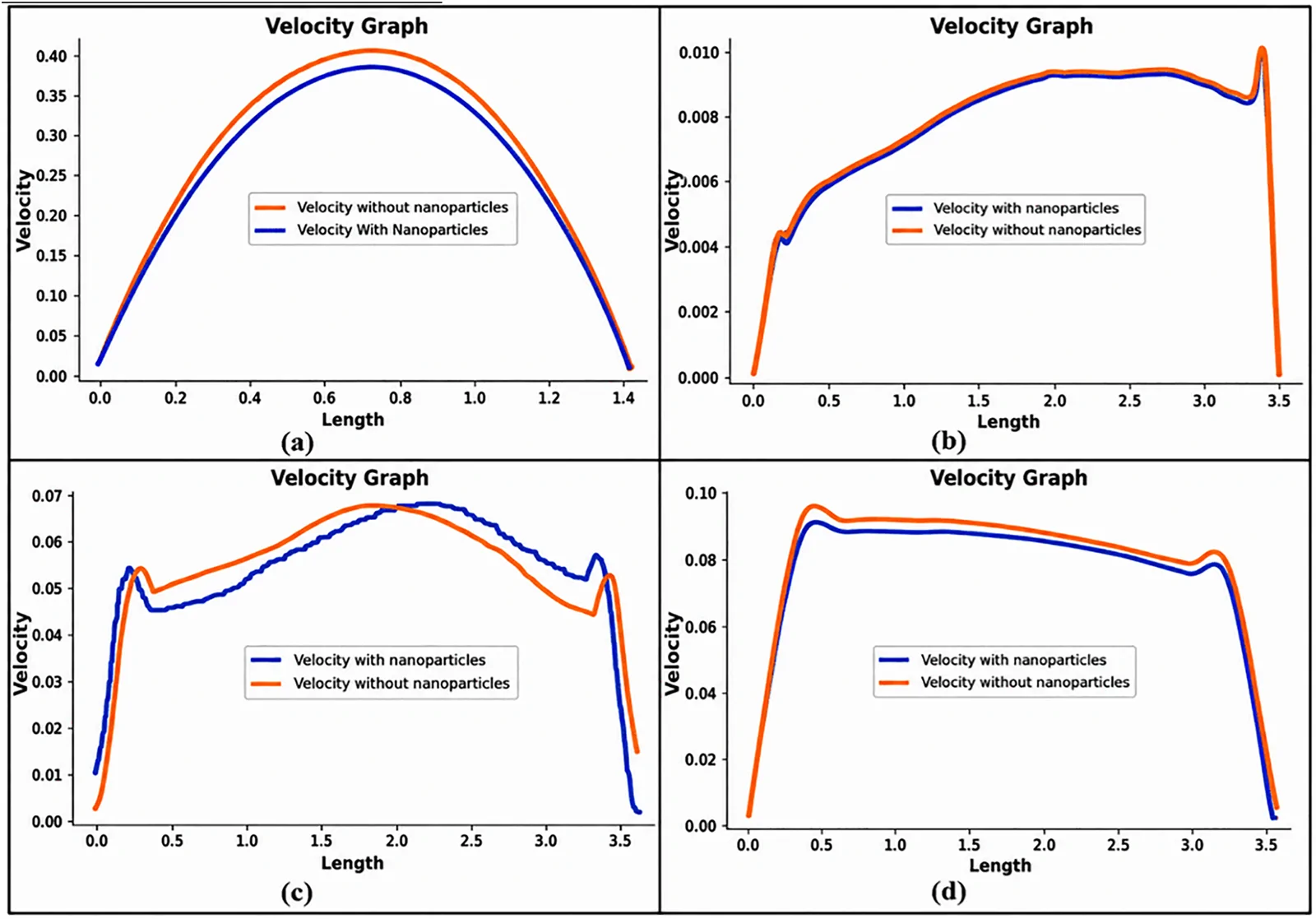
Velocity profiles for (a) healthy artery (Model 1) and (b – d) diseased arteries (Model 2 – Model 4).

In contrast to the healthy artery, where nanoparticles attenuate the peak velocity, their presence in the diseased artery promotes a modest but beneficial increase in local flow. Model 4 (Fig 9d) exhibits a right-skewed velocity distribution in the aneurysmal region, reflecting the strong influence of geometric asymmetry and vessel curvature. The interaction between curvature-induced secondary flow and aneurysmal expansion shifts the high-velocity core toward the outer region of the lumen.

These findings demonstrate that the hemodynamic influence of nanoparticles is highly geometry-dependent. While nanoparticle addition reduces peak velocity in the healthy artery, it enhances flow circulation and slightly increases velocity within aneurysmal sacs. Such behavior indicates that nanoparticles are particularly effective in disturbed flow environments, where they help mitigate recirculation and improve momentum exchange. Furthermore, the branching daughter vessels and arterial curvature play a crucial role in generating secondary flow structures. The streamwise velocity becomes distorted near the inner vessel wall, producing asymmetric velocity contours consistent with previously reported studies [63,64]. In the iliac branches, the velocity core shifts toward the inner wall in the x-y plane (Figs 9b-9d), leaving low-momentum regions adjacent to the wall. These regions subsequently evolve into localized recirculation zones, a hallmark of complex arterial flow in curved and bifurcated geometries.

### Pressure difference in healthy and diseases AAA

Hemodynamic pressure distributions provide critical insight into the flow behavior of nanoparticle-enriched blood within diseased abdominal arteries, revealing the complex interactions among vascular geometry, fluid properties, and local biomechanical forces.

Fig 10a-10d depicts the pressure distributions for all investigated abdominal aortic models under both conventional blood flow and nanoparticle-enhanced conditions. In all cases, the incorporation of nanoparticles leads to a noticeable increase in pressure, primarily due to the higher effective density and viscosity of the nanofluid, which elevate the overall flow resistance. For the healthy artery (Model 1), the maximum pressure rises from 1410 Pa to 1857.4 Pa following nanoparticle addition. In the aneurysmal cases, substantially greater pressure levels are observed. Model 2 exhibits an increase from 9553 Pa to 10,640 Pa, while Model 3 records the highest pressure among all configurations, rising from 12,540 Pa to 13,737 Pa. Model 4, despite possessing the largest aneurysmal dilation, shows a comparatively lower peak pressure, increasing from 8730 Pa to 9298 Pa. A pronounced pressure gradient is evident near the inlet region in all diseased models, where the incoming flow undergoes rapid deceleration upon entering the expanded lumen. Although pressure generally increases with aneurysm size, Model 4 deviates from this trend. From a hemodynamic perspective, the pressure variation among Models 2–4 cannot be attributed solely to aneurysm size, as the local vascular configuration also governs flow redistribution and pressure recovery. Models 2 and 3 have the same overall vascular configuration, but Model 3 contains a larger aneurysmal dilation, resulting in a stronger modification of the local flow field and a higher-pressure level within the aneurysmal region. In contrast, although Model 4 possesses a larger primary aneurysm than Models 2 and 3, the presence of an additional aneurysmal dilation in the branching artery substantially alters the downstream flow distribution. The secondary aneurysm provides an additional region for flow expansion, redistribution, and energy dissipation, thereby modifying the pressure transmission within the parent artery. Consequently, the pressure in Model 4 is lower than that observed in Models 2 and 3 despite its larger primary aneurysm. These findings demonstrate that aneurysmal size alone does not determine the pressure response; rather, the combined effects of aneurysm morphology, branching configuration, flow redistribution, and local pressure gradients control the hemodynamic environment within the diseased artery. The relatively higher pressure observed in Model 3 therefore reflects its specific geometric configuration and flow response rather than aneurysm size alone. This behavior is likely associated with its complex geometric configuration, where enhanced flow redistribution, secondary vortices, and the interaction of multiple dilated regions reduce localized pressure accumulation.

**Fig 10:**
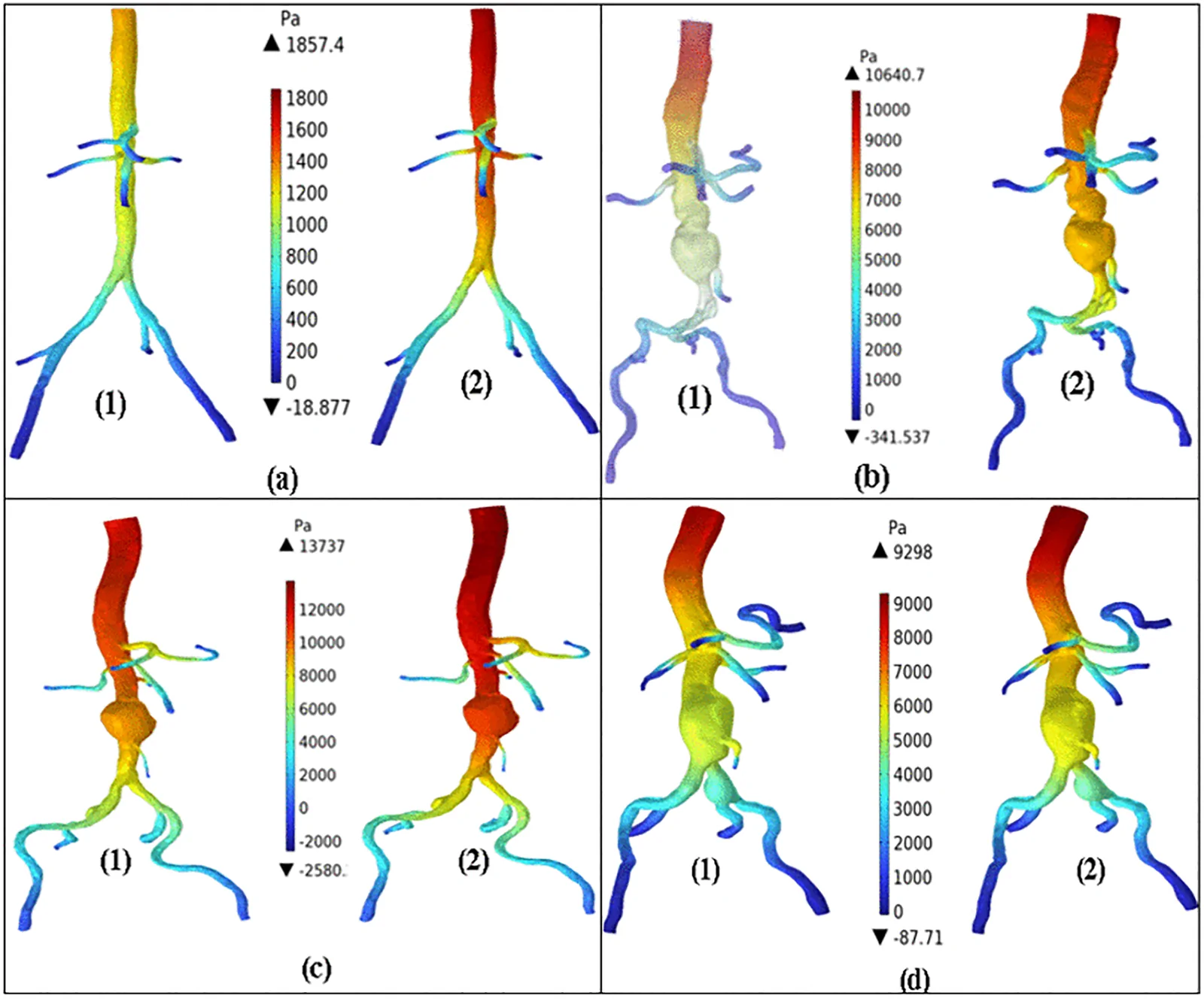
Volumetric pressure contour of different models: without nanoparticles- (a) 1, (b) 1, (c) 1, (d) 1; with nanoparticles- (a) 2, (b) 2, (c) 2, (d) 2.

In addition to aneurysm morphology, the branching daughter vessels and arterial curvature significantly influence the pressure field. These anatomical features generate secondary flow structures that alter momentum transport and produce asymmetric pressure distributions throughout the abdominal aorta. From a biomechanical standpoint, elevated intraluminal pressure directly increases circumferential wall tension, thereby accelerating aneurysm progression and increasing rupture susceptibility. As aneurysm diameter enlarges, the vessel wall is subjected to progressively greater hemodynamic loading, making size one of the most reliable predictors of rupture risk. The present results therefore underscore the critical role of aneurysm geometry in determining local pressure characteristics. Figs 11a-11d depicts the axial pressure distribution along the arterial arc length for all geometrical configurations in the presence of nanoparticles. The addition of nanoparticles leads to a noticeable rise in peak pressure across all models, reflecting their significant influence on the hemodynamic characteristics of blood flow. This increase is primarily associated with the enhanced thermal conductivity and modified transport properties of the blood–nanoparticle mixture, which contribute to greater flow stability and diminished local pressure oscillations. Models 2 and 3 exhibit a nearly uniform pressure augmentation throughout the arterial length, indicating a consistent hemodynamic response under nanoparticle loading. In contrast, Model 4, despite possessing the largest aneurysmal enlargement, maintains relatively lower pressure levels along much of the domain. The substantial lumen expansion promotes pressure dissipation, thereby moderating the axial pressure gradient. Meanwhile, the healthy artery (Model 1) displays the steeper pressure decline, a consequence of its narrower geometry and correspondingly higher hydraulic resistance. A further notable observation is the beneficial effect of nanoparticles on pressure recovery in Models 2, 3, and 4. Although the overall flow structure remains largely unchanged, localized improvements in flow transport are evident, with slight increases in velocity observed in selected regions compared with the nanoparticle-free case. This behavior suggests that nanoparticle incorporation enhances momentum exchange while preserving the fundamental flow characteristics. Collectively, these results demonstrate the intricate coupling between vascular geometry, blood rheology, flow dynamics, and nanoparticle transport. Such insights are essential for accurately characterizing aneurysmal hemodynamics and may aid in the design of nanoparticle-assisted therapeutic interventions aimed at improving cardiovascular performance while minimizing hemodynamic risk.

**Fig 11:**
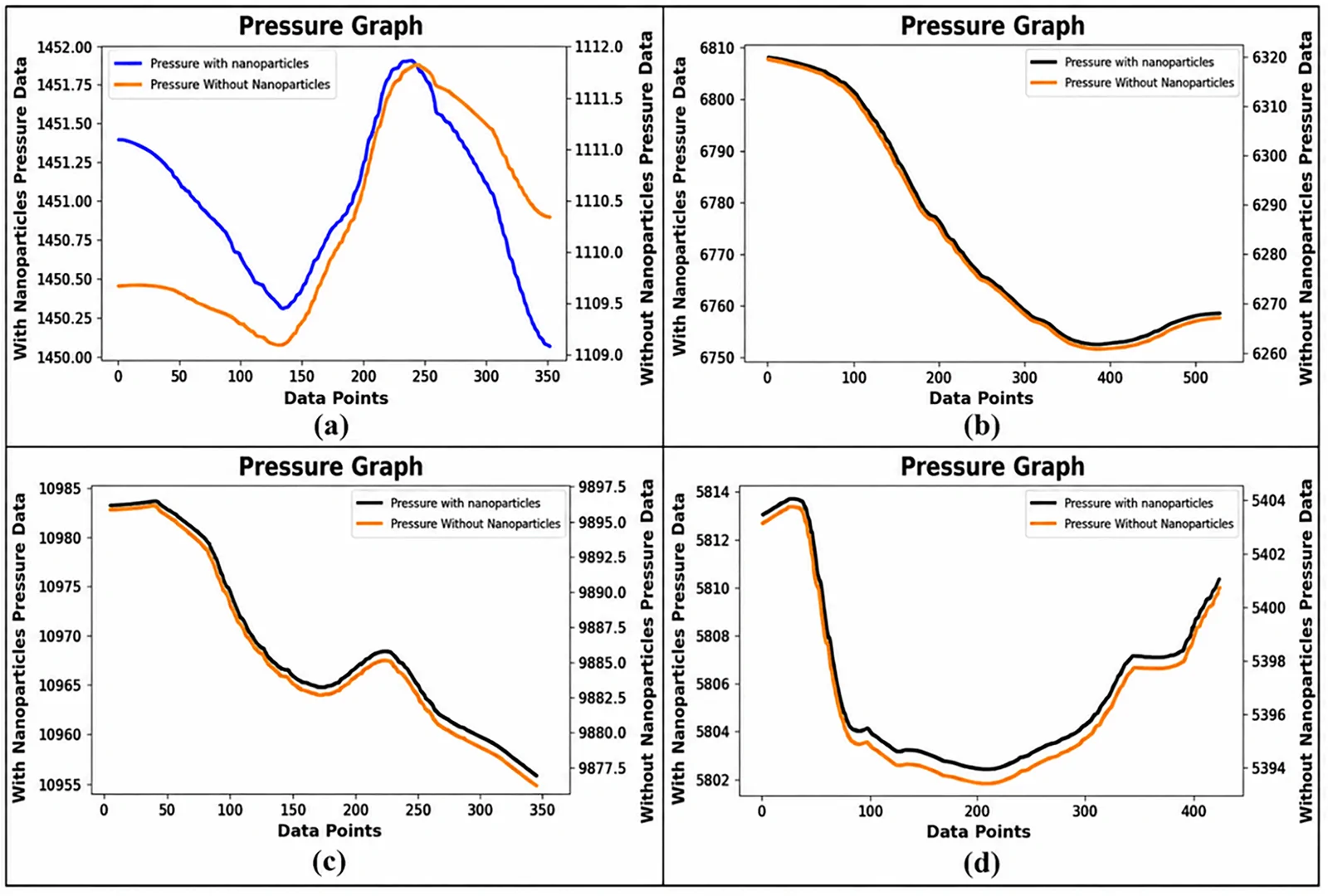
Pressure profiles for (a) healthy artery (Model 1) and (b – d) diseased arteries (Models 2 – Model 4).

### WSS distribution in different healthy and diseased AAA models

The distribution of WSS affects how blood and nanoparticles interact in patient specific abdominal arteries with aneurysm. Fig 12(1, 2(a-d)) compares the WSS distributions for all arterial models under nanoparticle-enhanced and nanoparticle-free conditions. In every case, nanoparticle incorporation results in higher WSS, indicating enhanced momentum transfer near the vessel wall. The healthy configuration exhibits the largest WSS, reaching approximately 64.8 Pa in the bifurcation and daughter branches, where blood acceleration is greatest. Without nanoparticles, the corresponding peak value decreases to 42.3 Pa. Aneurysmal geometries display a marked reduction in WSS relative to the healthy artery, primarily due to flow deceleration, separation, and the formation of recirculation zones within the dilated region. Model 2 records the lowest WSS, approximately 0.285 Pa, whereas Models 3 and 4 show moderately higher values of 1.04 Pa and 0.663 Pa, respectively. Under nanoparticle-free conditions, the corresponding values are 0.15 Pa, 0.50 Pa, and 0.40 Pa. The WSS response differs among Models 2–4 because of their distinct aneurysmal configurations and the resulting flow redistribution. Models 2 and 3 share the same vascular topology, but Model 3 contains a larger aneurysmal dilation. The increased geometric disturbance in Model 3 produces stronger local flow acceleration and steeper near-wall velocity gradients, particularly around the aneurysm neck and transition regions, resulting in higher localized WSS than in Model 2. In Model 2, the smaller aneurysm causes a comparatively weaker disturbance of the flow field and therefore produces lower peak WSS. Although Model 4 contains a larger primary aneurysm than Models 2 and 3, its additional aneurysmal dilation in the branching artery substantially modifies the downstream flow distribution. The incoming flow is divided and redistributed between the parent and branching vessels, while the enlarged regions provide greater opportunity for flow expansion, recirculation, and energy dissipation. Consequently, the near-wall velocity gradients are reduced in key regions, leading to a lower peak WSS than that observed in Model 3. Thus, the WSS trend among Models 2–4 is governed not simply by aneurysm size, but by the combined effects of aneurysm morphology, branching configuration, flow acceleration, and recirculation. Model 3 exhibits the strongest localized wall shear loading because its geometry promotes a greater near-wall velocity gradient, whereas the additional aneurysmal branch in Model 4 redistributes the flow and moderates the local WSS. These variations reflect the combined influence of aneurysm size, lumen expansion, and downstream branching complexity on local shear characteristics. Regions exposed to low WSS (<0.4 Pa) are of particular clinical importance, as prolonged shear deprivation has been strongly associated with endothelial dysfunction [65], inflammatory activation [66,67], and progressive vascular remodeling [68]. Such conditions are widely recognized as key contributors to aneurysm initiation, enlargement, and eventual rupture. The present results demonstrate that nanoparticle addition substantially elevates WSS throughout the diseased segments, thereby partially mitigating these low-shear environments. Overall, the enhancement in WSS is especially evident in larger aneurysms, suggesting that nanoparticle transport may improve near-wall hemodynamics and reduce adverse mechanical conditions. These findings highlight the potential of nanoparticle-assisted flow modulation as a promising strategy for aneurysm stabilization and rupture risk reduction.

**Fig 12:**
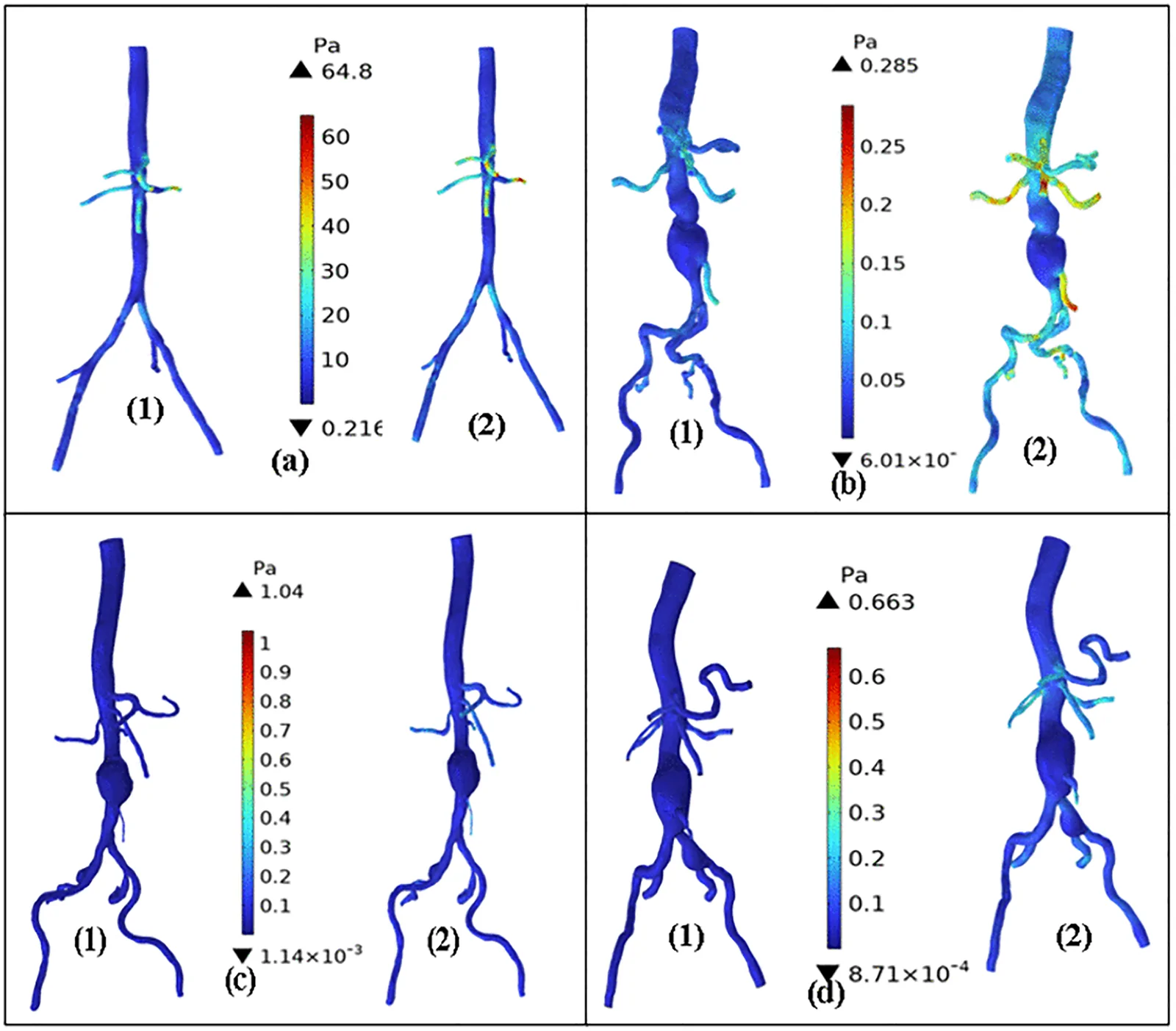
Volumetric WSS contour of different models: Without nanoparticles- (a) 1, (b) 1, (c) 1, (d) 1; with nanoparticles- (a) 2, (b) 2, (c) 2, (d) 2.

Fig 13a-13d presents the WSS distribution for all configurations under both nanoparticle-enhanced and nanoparticle-free conditions. In the healthy artery (Model 1), the highest shear levels occur adjacent to the vessel wall, reaching approximately 6.0 Pa with nanoparticles and 4.25 Pa without nanoparticles. This elevated near-wall shear reflects the streamlined nature of the flow and the absence of significant recirculation. With the onset of aneurysmal dilation, WSS decreases substantially within the enlarged sac. Model 2 exhibits the lowest shear levels, ranging from 0.005 Pa to 0.017 Pa in the nanoparticle case and from 0.004 Pa to 0.012 Pa without nanoparticles. For Models 3 and 4, similarly low WSS is observed within the aneurysm core, although localized peaks develop near the aneurysm peripheral walls. Specifically, Model 3 attains values below 0.030 Pa with nanoparticles and below 0.016 Pa without nanoparticles, whereas Model 4 reaches approximately 0.036 Pa and 0.025 Pa, respectively. These distributions indicate a pronounced redistribution of hemodynamic forces: the aneurysm interior experiences persistent low shear due to flow stagnation and recirculation, while the surrounding wall regions are subjected to comparatively elevated stresses. Such a pattern is clinically significant, as sustained low WSS promotes endothelial dysfunction, inflammatory activity, and continued aneurysmal expansion. Conversely, localized high WSS near the aneurysm neck and wall may accelerate structural weakening, thereby increasing rupture susceptibility. The spatial heterogeneity of WSS demonstrates that aneurysmal geometry redistributes wall loading, creating regions of both abnormally low and elevated shear that can influence endothelial function, vascular remodeling, and the mechanical stability of the arterial wall.

**Fig 13:**
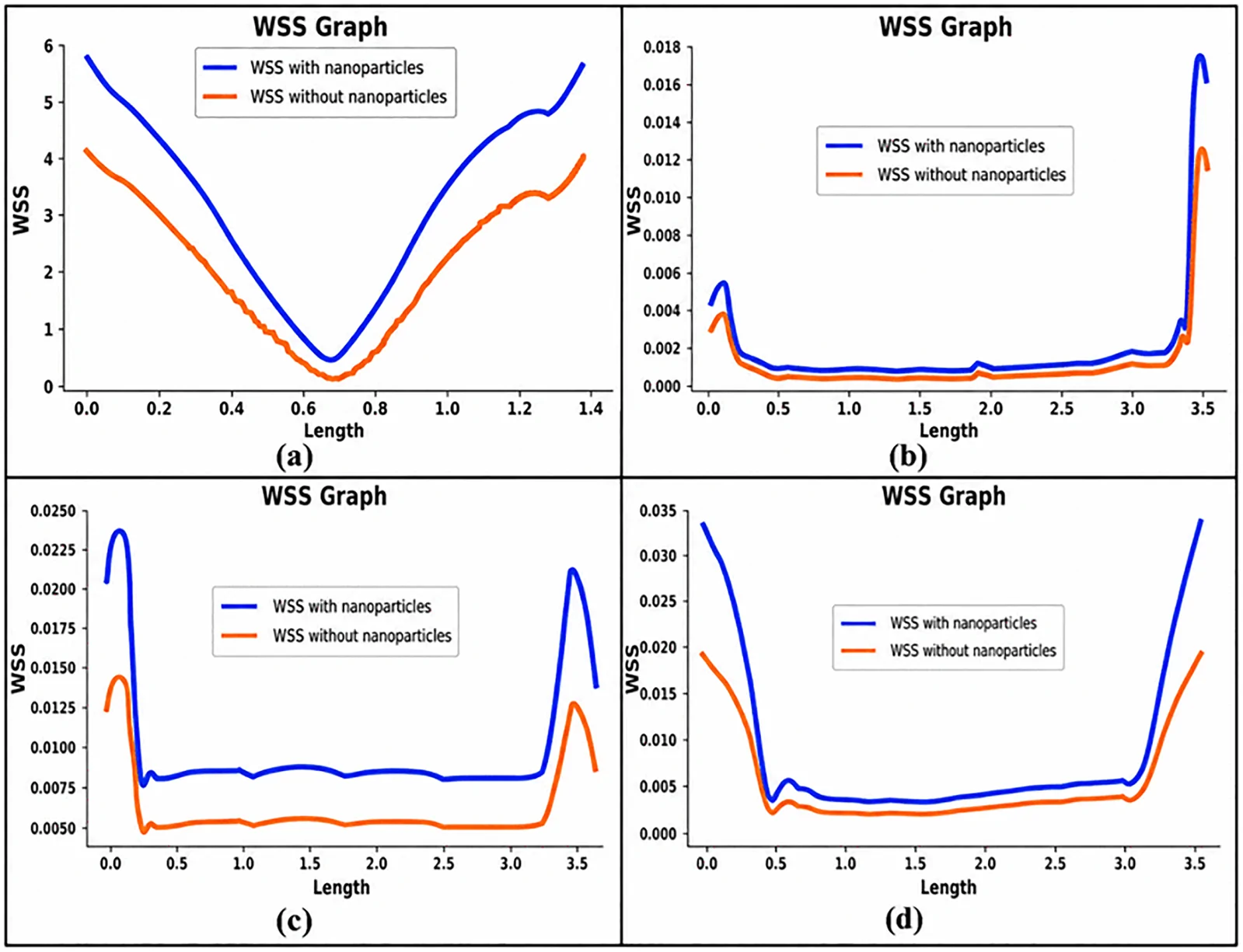
WSS profiles for (a) healthy artery (Model 1) and (b–d) diseased arteries (Models 2–4).

The nanoparticle Ag incorporation consistently elevates WSS throughout the domain while preserving the characteristic shear distribution. This enhancement suggests improved near-wall momentum transport and may offer potential benefits for modulating adverse hemodynamic conditions in aneurysmal arteries.

### ANN topology optimization in different healthy and diseased AAA models

A triple-hidden-layer ANN based on the recurrent backpropagation algorithm is developed to investigate the simulation data obtained from the present computational study. The numerically generated dataset served as the input for network training and subsequent validation. The numerically obtained CFD dataset was used for network training and subsequent validation. In the adopted feedforward architecture, information is transmitted sequentially from the input layer through the hidden layers to the output layer, with no recurrent or feedback connections. The adopted network architecture (Fig 14) comprises three hidden layers containing five, four, and three neurons, respectively. Such a configuration provides sufficient flexibility to capture the highly nonlinear relationship between the governing input parameters and the associated hemodynamic responses in aneurysmal abdominal arteries. During the development of the ANN models, particular attention was given to achieving reliable generalization beyond the training data. Overfitting may occur when an ANN captures the specific characteristics of the training dataset rather than the underlying flow relationships, resulting in reduced predictive accuracy for unseen data [69,70]. To minimize this effect, model selection was based on two criteria: low prediction errors for both the training and test datasets and a small difference between their respective errors. The number of neurons in the hidden layer was therefore selected through an iterative trial-and-error procedure, with the final architecture chosen based on its overall predictive performance and generalization capability. To ensure robust model generalization, the complete dataset is randomly partitioned into training (60%), testing (20%), and cross-validation (20%) subsets. A standard feedforward multilayer perceptron (MLP) was employed, in which information propagates sequentially from the input layer through the hidden layer(s) to the output layer without recurrent or feedback connections. The network was trained over 500 epochs using a momentum coefficient of 0.70. Model performance is assessed using the mean squared error (MSE), which demonstrate excellent predictive capability. The minimum MSE achieved is 8.81 × 1^−4^ for the training dataset, while the corresponding cross-validation error is 5.43 × 10^−3^. These low error values confirm the stability, accuracy, and generalization capability of the proposed ANN model.

**Fig 14:**
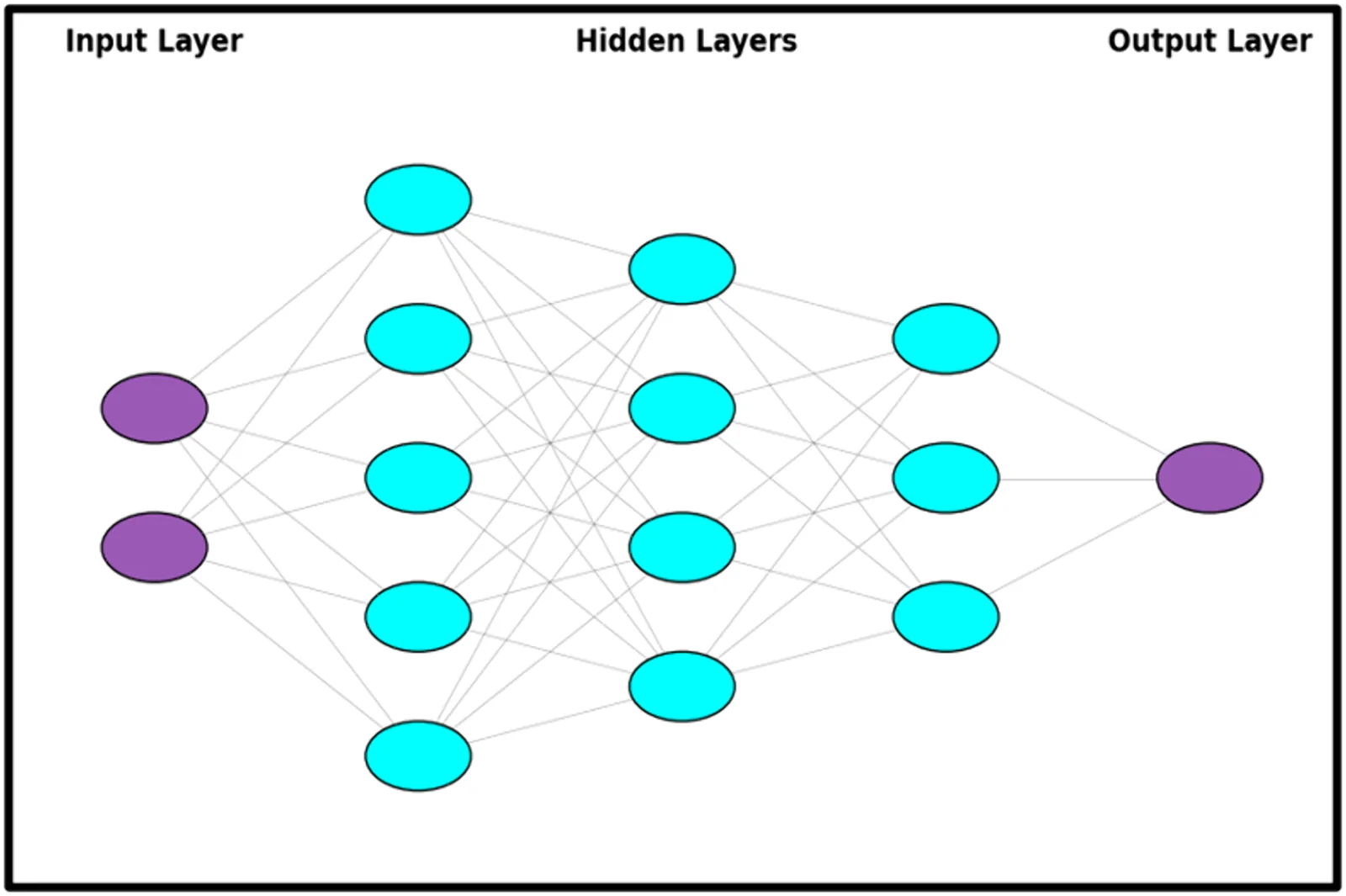
Three-layer neural network frame for simulating and predicting efficiency.

The predictive performance across the training, testing, cross-validation, and independent statistical validation datasets is illustrated in Figs 15 and 16.

**Fig 15:**
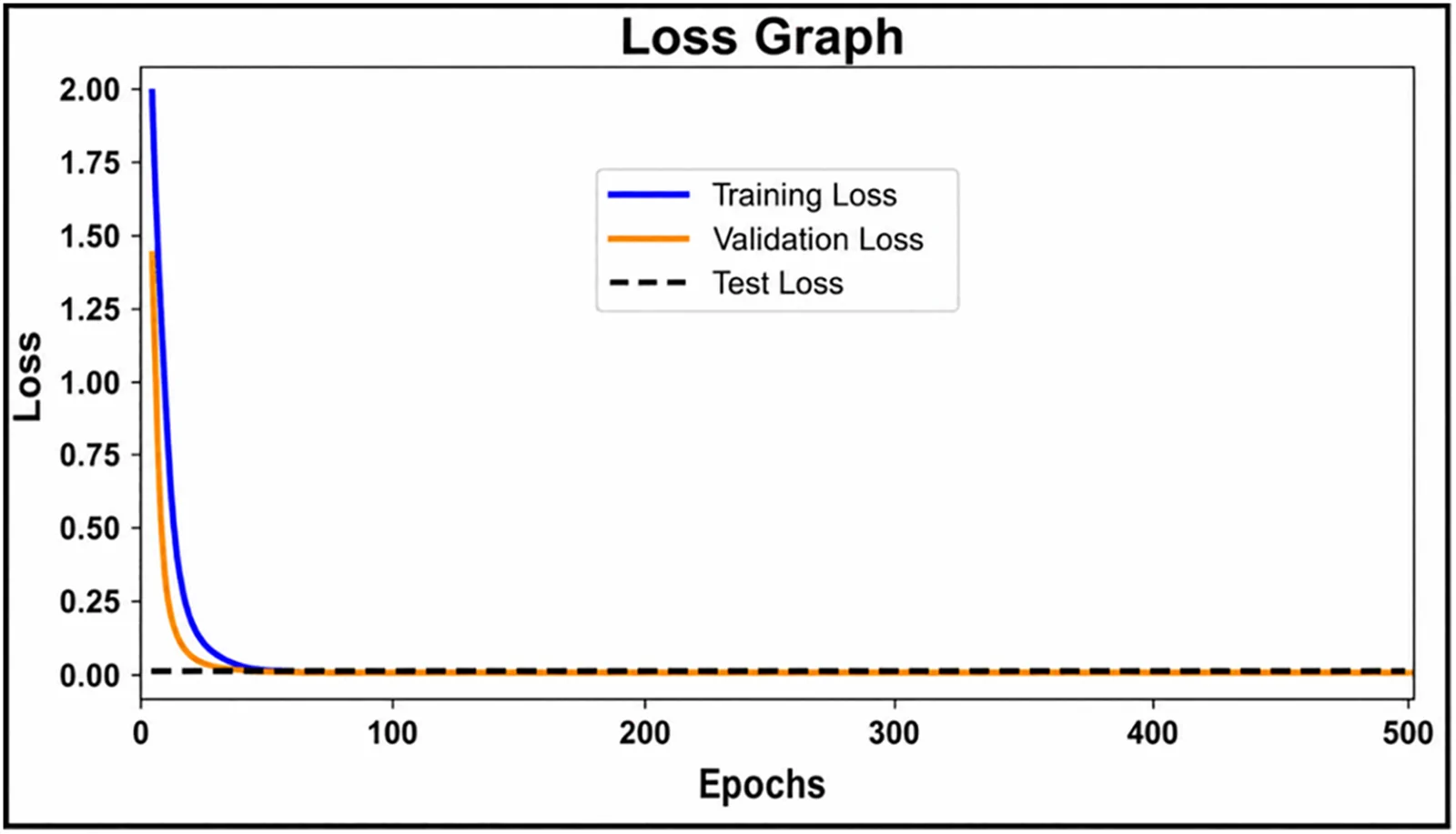
MSE convergence profile over 500 training epochs.

**Fig 16:**
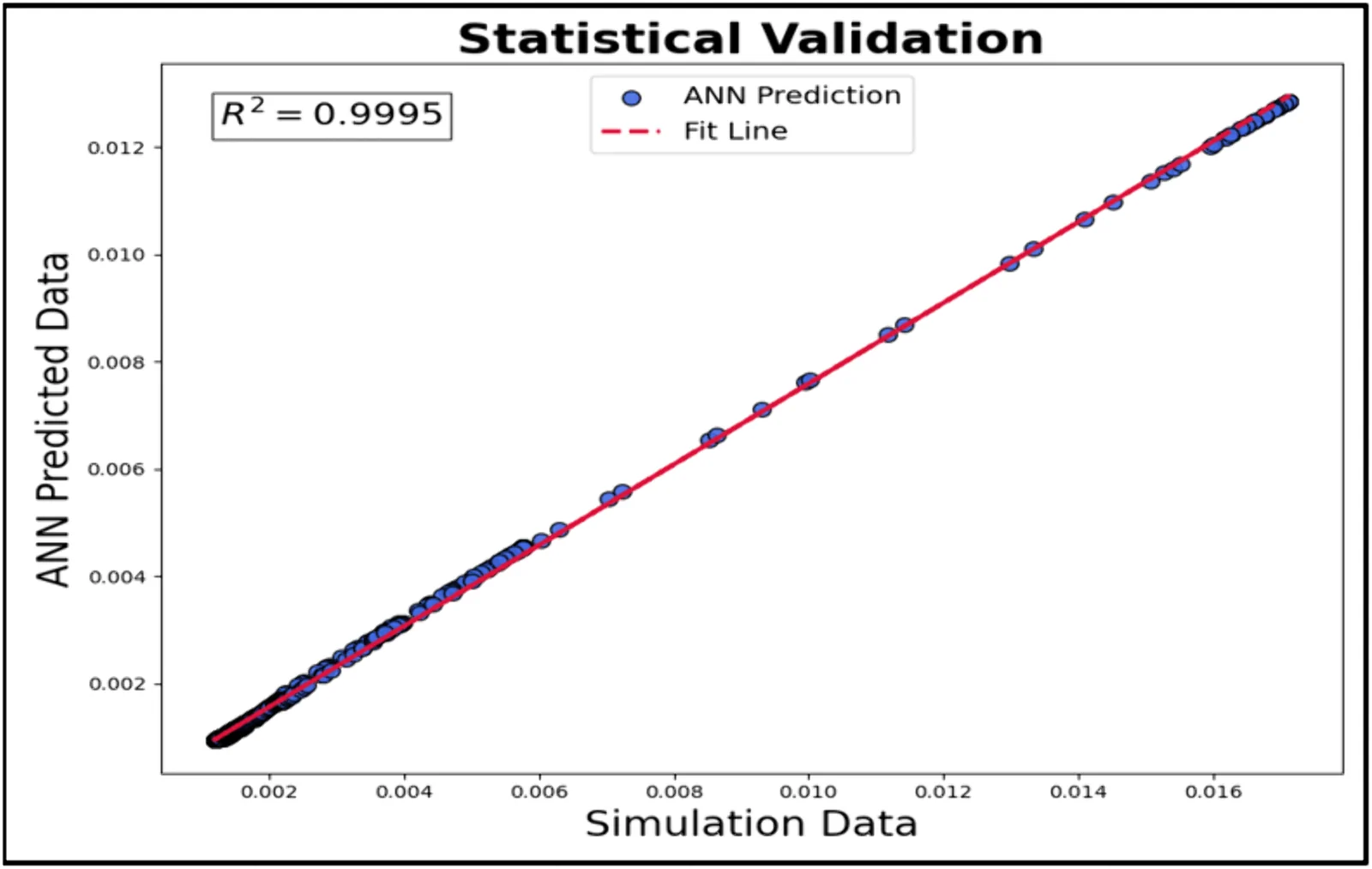
Regression result of simulation data.

Fig 15 illustrates the convergence history of the ANN during the training process over 500 epochs. The training loss and validation loss decrease rapidly during the initial epochs, indicating efficient learning and fast optimization of the network parameters. As the training progresses, both curves continue to decline gradually and eventually stabilize near zero, demonstrating excellent convergence behavior and numerical stability. The close agreement between the training and validation loss curves throughout the learning process indicates that the developed ANN model generalizes well and does not suffer from overfitting. Additionally, the test loss remains consistently very low across all epochs, further confirming the robustness, predictive capability, and reliability of the trained model. Overall, the convergence characteristics demonstrate that the ANN has achieved high accuracy and strong generalization performance, making it highly suitable for predicting the underlying physical phenomena.

Fig 16 presents the statistical validation between the simulation results and the corresponding predicted values. The data points closely follow the 45° reference line, indicating excellent agreement across the entire range of observations. This strong linear correlation demonstrates the high predictive capability and robustness of the developed model. The minimal scatter around the regression line confirms the low prediction error and the reliability of the computational framework. Both low- and high-magnitude values are accurately captured, suggesting that the model maintains consistent performance throughout the dataset. Such close agreement verifies that the predictive approach effectively reproduces the underlying hemodynamic behavior obtained from the numerical simulations. Overall, the validation results confirm the accuracy, stability, and generalization capability of the proposed methodology, supporting its suitability for reliable hemodynamic prediction and further biomedical flow analyzes.

The effects of key hemodynamic parameters, namely velocity, pressure, and wall shear stress, are systematically investigated in abdominal arteries with varying aneurysm severities under both conventional and nanoparticle-enhanced blood flow conditions. The resulting flow characteristics are compared with those of a healthy arterial configuration to quantify the influence of aneurysmal deformation. To complement the computational analysis, an ANN is employed to predict the hemodynamic responses and to verify the consistency of the numerical simulations. The ANN successfully captures the nonlinear relationship between the investigated flow conditions and the resulting hemodynamic responses. From a physical perspective, the consistency between the CFD and ANN results supports the reliability of the numerical predictions and demonstrates the potential of the ANN as a computationally efficient surrogate for estimating hemodynamic quantities. Collectively, these findings strengthen the proposed hybrid CFD–ANN framework for analyzing the hemodynamic effects of aneurysmal severity and nanoparticle-enhanced blood flow. As illustrated in Figs 17–19a-d, good agreement is observed between the CFD results and the ANN predictions [71], confirming the robustness, predictive capability, and reliability of the proposed hybrid computational framework.

**Fig 17:**
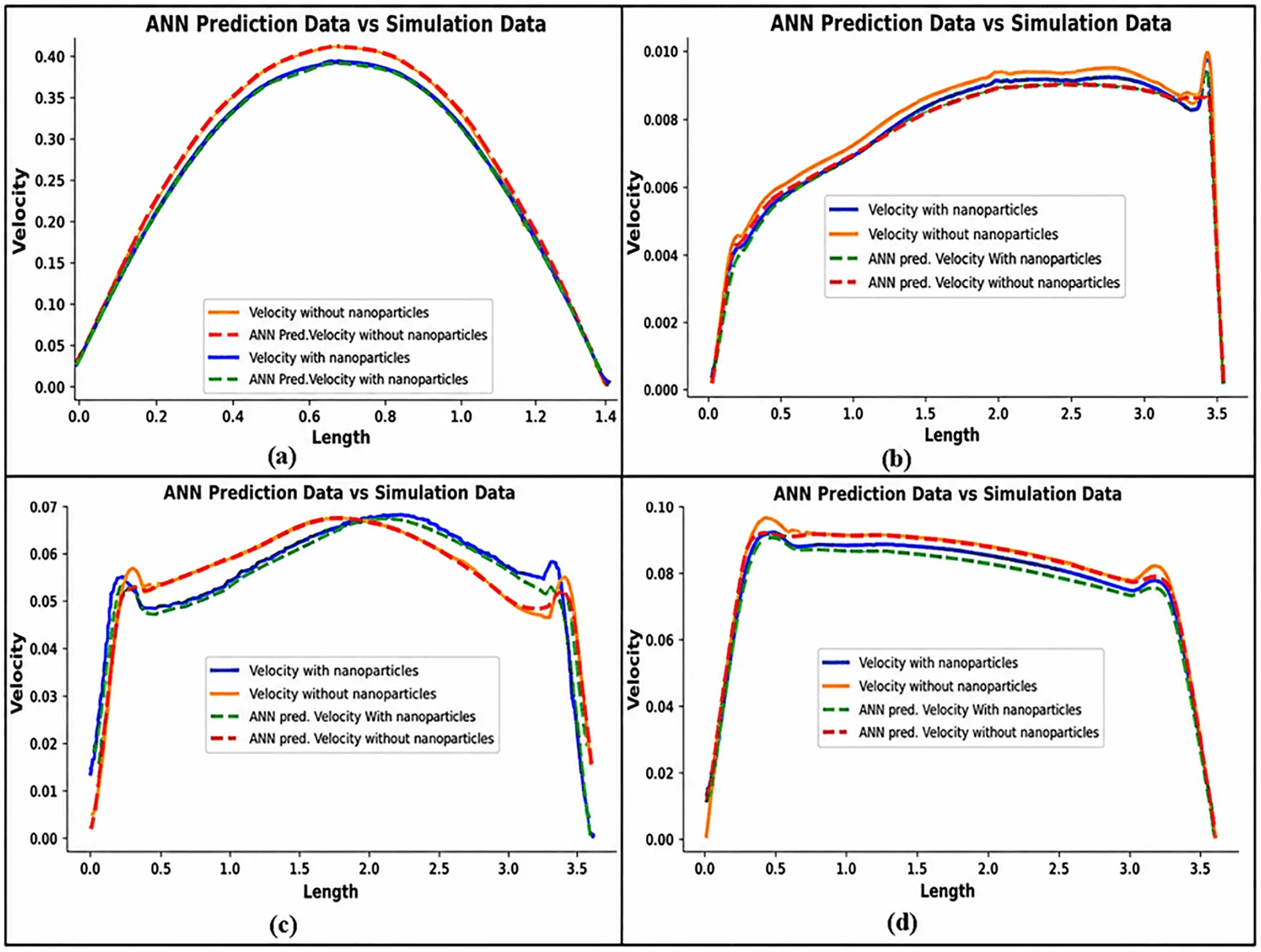
Velocity profiles for (a) healthy artery (Model 1) and (b–d) diseased arteries (Models 2–4) with ANN prediction.

**Fig 18:**
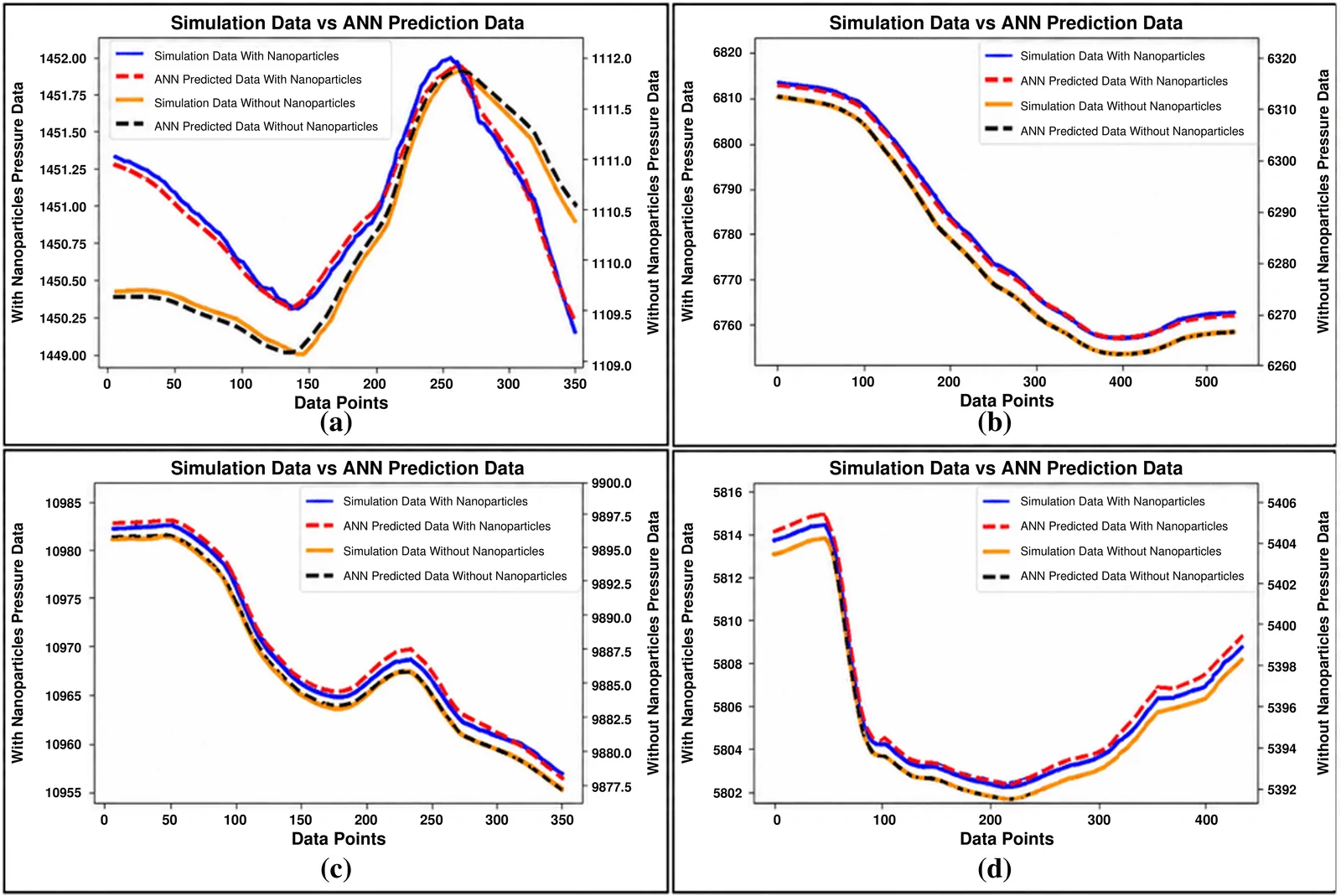
Pressure profiles for (a) healthy artery (Model 1) and (b–d) diseased arteries (Models 2–4) with ANN prediction.

**Fig 19:**
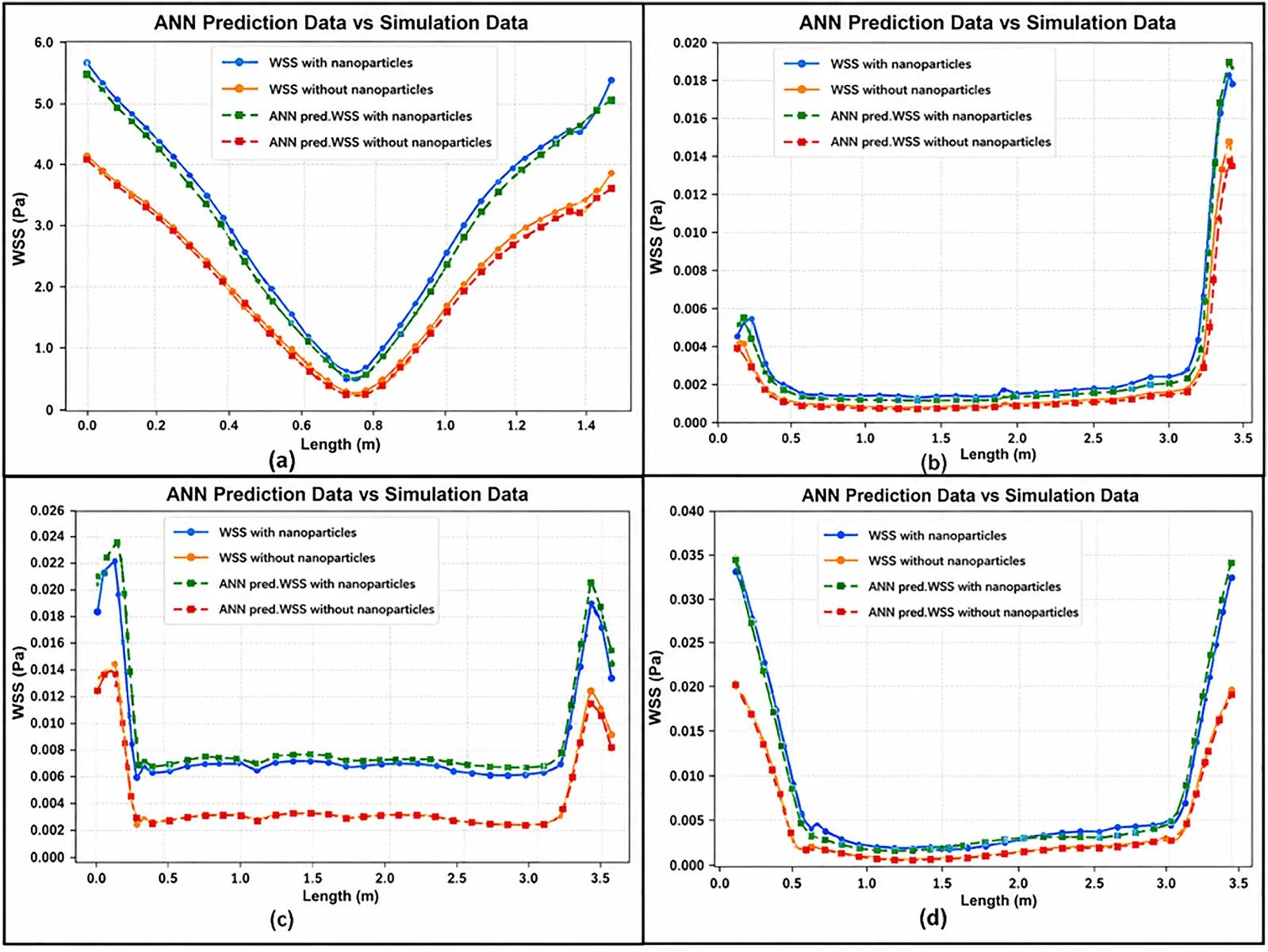
WSS profiles for (a) healthy artery (Model 1) and (b–d) diseased arteries (Models 2–4) with ANN prediction.

### Comparison with published results

Our simulation of the abdominal aortic artery with aneurysm revealed the highest blood velocity and wall shear stress. These results are consistent with Müftüoğulları et al.[72], who showed that bifurcation angles have a significant impact on flow patterns, wall shear stress, and oscillatory shear index (OSI). Ghandi et al. [73] found that nanoparticles help in controlling wall shear stress and flow dynamics. In our study, adding nanoparticles increased arterial pressure by 5.22% –10.22%, enhancing hemodynamic conditions. Similar effects, depending on the type and concentration of nanoparticles, were also noted by Fetuga et al. [74], which matches our findings in complex artery geometries. Additionally, Doutel et al. [75] stated that irregular artery shapes cause secondary flows, such as recirculation and vortices, in low wall shear stress areas, and we observed similar flow behavior in branching regions. Wahad et al. [76] showed that the shape of the artery and its aneurysm characteristics affect pressure and velocity distributions. Our bifurcated artery models showed similar trends, reinforcing the importance of vascular geometry on blood flow.

## Conclusions

This study investigated blood flow in a three-dimensional patient-specific abdominal artery with an aneurysm using a non-Newtonian model, silver nanoparticles, and ANN based prediction. The results demonstrate that blood rheology and nanoparticle incorporation significantly affect velocity, pressure, and wall shear stress, while the ANN accurately predicts the hemodynamic behavior. The key findings are summarized below:

1) Model 3 exhibits the velocity increased (59.08%), followed by Models 2 (52.59%) and 4 (40.21%) compare to model 1, confirming the influence of aneurysm on arterial flow dynamics. These hemodynamic alternations modify the mechanical environment of the arterial wall and may contribute to vascular remodeling and disease progression.
2) In the healthy artery (Model 1), nanoparticle incorporation reduces the peak velocity from 0.41 to 0.37 m/s, representing a 9.76% decrease. Models 2 and 4 exhibit identical velocity, while Model 3 shows a velocity increase of 7.35%. Collectively, these results demonstrate that nanoparticle addition enhances flow uniformity and promotes hemodynamic stability within aneurysmal arteries. Such velocity alterations can modify near-wall velocity gradients and the distribution of wall shear stress and pressure, which are important mechanical factors associated with endothelial dysfunction and vascular remodeling.
3) Nanoparticle incorporation increases arterial pressure across all investigated models. In Model 1, the peak pressure rises from 1410 to 1857 Pa, corresponding to a 24.07% increase. Similar enhancements are observed in Models 2, 3, and 4, with increases of 10.22%, 8.70%, and 6.11%, respectively. Overall, these findings indicate that nanoparticle addition improves pressure distribution and contributes to enhanced hemodynamic stability.
4) Nanoparticle incorporation increases arterial pressure also at an aneurysmal region in all models, with enhancements of 23.45%, 7.27%, 9.86%, and 7.04% for Models 1–4, respectively, thereby improving pressure distribution. Therefore, the pressure distribution, together with WSS and velocity alterations, provides an important indicator for evaluating the hemodynamic environment associated with aneurysmal arterial disease.
5) Nanoparticle incorporation leads to a consistent increase in wall shear stress (WSS) across all examined configurations. In the healthy artery (Model 1), WSS increases from 42.3 to 64.8 Pa, corresponding to a 34.72% rise. Models 2, 3, and 4 exhibit enhancements of 47.37%, 51.92%, and 39.67%, respectively. These results emphasize the significance of incorporating non-Newtonian blood behavior to achieve accurate WSS estimation, which is essential for understanding endothelial loading and potential disease progression.
6) In all aneurysmal models, nanoparticle incorporation consistently increases the evaluated hemodynamic parameters relative to the base-fluid condition.
7) The velocity, pressure and WSS values predicted by the artificial neural network exhibited excellent agreement with the numerical simulation results, confirming the robustness, accuracy, and predictive reliability of the developed model.

The present study demonstrates that the integration of nanoparticle-assisted hemodynamics, non-Newtonian blood rheology, and artificial neural network modeling provides a powerful framework for analyzing flow in diseased abdominal arteries. The non-Newtonian formulation captures the complex rheological behavior of blood more realistically, while nanoparticle suspension enhances transport characteristics and may improve targeted therapeutic delivery. In addition, the artificial neural network accurately identifies hemodynamic patterns and enables rapid, non-invasive assessment of disease severity. The minimal dataset supporting the findings of this current study is provided as S1 Dataset. Collectively, these approaches establish an efficient and reliable computational strategy for advancing nanoparticle-based vascular therapies and supporting clinical decision-making in the management of abdominal arterial disorders.

### Limitations and future works

Several limitations should be acknowledged. The blood was modeled using a simplified non-Newtonian formulation, which cannot fully represent the complex rheological behavior of blood under all physiological conditions. Although patient-specific arterial geometries were considered, the vessel walls were treated as rigid and fluid–structure interaction (FSI) was not included. The nanoparticle analysis was also limited to silver (Ag) nanoparticles; therefore, the findings cannot be directly generalized to other nanoparticle types, such as gold or magnetic nanoparticles, who’s physical and transport properties may differ considerably. Furthermore, temperature effects on blood rheology and nanoparticle transport were not considered. These factors may influence nanoparticle migration and local flow characteristics.

Future studies incorporating compliant arterial walls, FSI, thermal effects, alternative nanoparticle materials, and coupled energy transport would provide a more comprehensive representation of nanoparticle-assisted blood flow under physiological conditions.

### Nomenclature

**Table:** 

AAA	Abdominal aortic aneurysm	
CFD	Computational fluid dynamics	
WP	Wall pressure	
WSS	Wall shear stress	
TAWSS	Time average WSS	
OSI	Oscillatory shear index	
RRT	Relative residence time	
MCFD	Multiphase computational fluid dynamics	
SMA	Superior mesenteric artery	
IMA	Inferior mesenteric artery	
FEM	Finite element method	
UDF	User defined function	
ANN	Artificial neural network	
MSE	Mean squared error	
T	Period of the cardiac cycle	**[s]**
τ	Wall shear stress	**[Pa]**
μ	Dynamic viscosity	**[Pa. s]**
u	Blood velocity	**[m/s]**
∇u	Gradient velocity vector	**[1/s]**
F	Body force	**[kg/m** ^**3**^ **s** ^**2**^ **]**
λ	Relaxation time	**[s]**
$\mu_{0}$	Zero shear rate viscosity	**[Pa. s]**
$\mu_{\infty }$	Infinite shear rate viscosity	**[Pa. s]**
n	Power index	**Dimensionless**
a	Transition parameter	**Dimensionless**
α	Pressure amplitude	**[Pa]**
I	Identity vector	**Dimensionless**
P(t)	Blood pressure	**[Pa]**
v	Kinematic viscosity	**[m** ^**2**^ **/s]**

## Supporting information

S1 Dataset Minimal dataset supporting the findings of this study. (DOCX)
