# Supplementary material for: Computational investigation and machine learning prediction of nanoparticle effects on hemodynamics in patient-specific aortic aneurysms
Source: PLoS One. 2026 Oct 7;21(10):e0350224. doi: 10.1371/journal.pone.0350224 (PMC13645084; doi:10.1371/journal.pone.0350224)
Supplement: Supplementary file 1 [file pone.0350224.s001.docx]

S1 Dataset: Minimal dataset supporting the findings of this study

| WSS | |
| --- | --- |
| Without nanoparticle | With nanoparticle |
| 4.195544 | 5.78059 |
| 4.185159 | 5.767386 |
| 4.179631 | 5.76035 |
| 4.170594 | 5.748837 |
| 4.133186 | 5.701038 |
| 4.124853 | 5.690357 |
| 4.119053 | 5.682915 |
| 4.1065 | 5.66679 |
| 4.073087 | 5.623723 |
| 4.06672 | 5.615492 |
| 4.061 | 5.608089 |
| 4.044952 | 5.587285 |
| 4.015331 | 5.54874 |
| 4.010837 | 5.542874 |
| 4.005646 | 5.536092 |
| 3.986043 | 5.510424 |
| 3.960008 | 5.476185 |
| 3.957282 | 5.47259 |
| 3.953218 | 5.467225 |
| 3.929871 | 5.436316 |
| 3.907212 | 5.406163 |
| 3.906141 | 5.404732 |
| 3.90405 | 5.401941 |
| 3.876542 | 5.365074 |
| 3.872313 | 5.359385 |
| 3.85795 | 5.340003 |
| 3.857494 | 5.339386 |
| 3.857044 | 5.338779 |
| 3.826151 | 5.296796 |
| 3.81287 | 5.27862 |
| 3.811427 | 5.276641 |
| 3.80961 | 5.274146 |
| 3.77883 | 5.231625 |
| 3.769927 | 5.21923 |
| 3.768057 | 5.216621 |
| 3.765023 | 5.212382 |
| 3.734704 | 5.169691 |
| 3.729411 | 5.162173 |
| 3.727487 | 5.159434 |
| 3.723402 | 5.15361 |
| 3.693905 | 5.11113 |
| 3.691562 | 5.107722 |
| 3.689827 | 5.105195 |
| 3.684874 | 5.09796 |
| 3.656552 | 5.052631 |
| 3.655566 | 5.051088 |
| 3.652606 | 5.04645 |
| 3.649566 | 5.041721 |
| 3.64397 | 5.033063 |
| 3.618923 | 4.994929 |
| 3.616665 | 4.991545 |
| 3.611173 | 4.983351 |
| 3.581699 | 4.940131 |
| 3.579396 | 4.936807 |
| 3.573385 | 4.928159 |
| 3.570741 | 4.924372 |
| 3.569679 | 4.922853 |
| 3.536969 | 4.876717 |
| 3.526735 | 4.862529 |
| 3.52669 | 4.862467 |
| 3.525065 | 4.860227 |
| 3.494679 | 4.818821 |
| 3.491633 | 4.814665 |
| 3.477318 | 4.795478 |
| 3.476552 | 4.794453 |
| 3.475538 | 4.793097 |
| 3.443364 | 4.75064 |
| 3.426424 | 4.728609 |
| 3.422935 | 4.724093 |
| 3.419761 | 4.719993 |
| 3.392172 | 4.68467 |
| 3.372372 | 4.659628 |
| 3.365874 | 4.651458 |
| 3.361845 | 4.646404 |
| 3.338052 | 4.616767 |
| 3.315152 | 4.588544 |
| 3.305371 | 4.576568 |
| 3.301394 | 4.571711 |
| 3.281003 | 4.546947 |
| 3.254757 | 4.515371 |
| 3.241427 | 4.499446 |
| 3.238193 | 4.495593 |
| 3.221026 | 4.475227 |
| 3.191186 | 4.440126 |
| 3.151998 | 4.397484 |
| 3.151915 | 4.397417 |
| 3.151592 | 4.394508 |
| 3.076309 | 4.314355 |
| 3.073765 | 4.312385 |
| 3.00635 | 4.235538 |
| 2.990997 | 4.220346 |
| 2.961214 | 4.18483 |
| 2.886025 | 4.091585 |
| 2.870958 | 4.077426 |
| 2.851457 | 4.059255 |
| 2.851266 | 4.05675 |
| 2.85107 | 4.054142 |
| 2.841324 | 4.046936 |
| 2.824469 | 4.034779 |
| 2.777545 | 3.974948 |
| 2.750777 | 3.937439 |
| 2.711842 | 3.893166 |
| 2.701638 | 3.879534 |
| 2.652411 | 3.822009 |
| 2.649279 | 3.814474 |
| 2.646053 | 3.806617 |
| 2.596199 | 3.750313 |
| 2.580677 | 3.733392 |
| 2.54675 | 3.687832 |
| 2.466771 | 3.607359 |
| 2.460136 | 3.593443 |
| 2.449382 | 3.570651 |
| 2.447807 | 3.567592 |
| 2.445322 | 3.562577 |
| 2.44319 | 3.557514 |
| 2.436811 | 3.543804 |
| 2.434979 | 3.539611 |
| 2.427 | 3.521357 |
| 2.338627 | 3.423542 |
| 2.313278 | 3.395803 |
| 2.290664 | 3.362367 |
| 2.242475 | 3.305258 |
| 2.225248 | 3.271895 |
| 2.190866 | 3.213551 |
| 2.146155 | 3.179218 |
| 2.141977 | 3.175872 |
| 2.027418 | 3.025381 |
| 2.013311 | 3.008223 |
| 1.973969 | 2.961636 |
| 1.920527 | 2.888171 |
| 1.793974 | 2.716752 |
| 1.782808 | 2.700513 |
| 1.697177 | 2.587488 |
| 1.692745 | 2.582298 |
| 1.688101 | 2.447296 |
| 1.597764 | 2.420464 |
| 1.583395 | 2.364739 |
| 1.53827 | 2.339588 |
| 1.522485 | 2.213885 |
| 1.432346 | 2.148691 |
| 1.392456 | 2.129638 |
| 1.374593 | 2.104963 |
| 1.357796 | 1.988179 |
| 1.272442 | 1.90218 |
| 1.208999 | 1.88747 |
| 1.208649 | 1.878329 |
| 1.198815 | 1.816906 |
| 1.13112 | 1.770105 |
| 1.130747 | 1.659636 |
| 1.118236 | 1.654335 |
| 1.04579 | 1.636717 |
| 1.044439 | 1.559656 |
| 1.03337 | 1.448939 |
| 0.970049 | 1.416944 |
| 0.899155 | 1.396535 |
| 0.882498 | 1.356971 |
| 0.866244 | 1.246548 |
| 0.828452 | 1.172382 |
| 0.759705 | 1.167527 |
| 0.720879 | 1.162541 |
| 0.708967 | 1.133703 |
| 0.6945 | 1.078105 |
| 0.628946 | 1.044262 |
| 0.607262 | 0.980214 |
| 0.57051 | 0.950047 |
| 0.563961 | 0.919284 |
| 0.527967 | 0.846223 |
| 0.50122 | 0.813897 |
| 0.462538 | 0.78878 |
| 0.416697 | 0.735332 |
| 0.416135 | 0.705531 |
| 0.415886 | 0.692355 |
| 0.397212 | 0.659806 |
| 0.359866 | 0.621794 |
| 0.350233 | 0.620223 |
| 0.348976 | 0.556728 |
| 0.311755 | 0.530492 |
| 0.304469 | 0.52306 |
| 0.298236 | 0.51306 |
| 0.294423 | 0.517433 |
| 0.296119 | 0.521627 |
| 0.303321 | 0.530765 |
| 0.30437 | 0.540276 |
| 0.311618 | 0.585169 |
| 0.338413 | 0.630399 |
| 0.357121 | 0.65182 |
| 0.364384 | 0.669965 |
| 0.371218 | 0.698859 |
| 0.383348 | 0.826583 |
| 0.457672 | 0.835475 |
| 0.462297 | 0.865547 |
| 0.472947 | 0.920231 |
| 0.514823 | 0.930886 |
| 0.526055 | 1.008115 |
| 0.571738 | 1.054607 |
| 0.61939 | 1.16734 |
| 0.674647 | 1.228463 |
| 0.719986 | 1.25 |
| 0.738118 | 1.300243 |
| 0.78519 | 1.431147 |
| 0.853316 | 1.506469 |
| 0.865425 | 1.548756 |
| 0.938821 | 1.55975 |
| 0.965849 | 1.604896 |
| 1.047098 | 1.644972 |
| 1.08297 | 1.709335 |
| 1.159418 | 1.82654 |
| 1.177778 | 1.832094 |
| 1.192682 | 1.887273 |
| 1.254374 | 1.889026 |
| 1.281826 | 2.001331 |
| 1.364975 | 2.117047 |
| 1.425179 | 2.208916 |
| 1.484188 | 2.309318 |
| 1.535335 | 2.408809 |
| 1.629675 | 2.520404 |
| 1.653631 | 2.545205 |
| 1.714068 | 2.609253 |
| 1.785947 | 2.677956 |
| 1.82907 | 2.713881 |
| 1.912942 | 2.876965 |
| 1.966515 | 2.964748 |
| 1.977603 | 2.970933 |
| 1.989812 | 2.977794 |
| 2.088773 | 3.130145 |
| 2.106861 | 3.146478 |
| 2.159357 | 3.207091 |
| 2.215033 | 3.284245 |
| 2.32654 | 3.430821 |
| 2.328474 | 3.435306 |
| 2.361538 | 3.478968 |
| 2.369144 | 3.480674 |
| 2.381994 | 3.498966 |
| 2.41655 | 3.530286 |
| 2.422116 | 3.545931 |
| 2.4351 | 3.579907 |
| 2.547862 | 3.707457 |
| 2.570865 | 3.733775 |
| 2.6173 | 3.800124 |
| 2.680117 | 3.875629 |
| 2.704432 | 3.901662 |
| 2.755999 | 3.962896 |
| 2.842839 | 4.071871 |
| 2.845688 | 4.071922 |
| 2.850877 | 4.072012 |
| 2.911849 | 4.07206 |
| 2.918809 | 4.14822 |
| 2.948196 | 4.157064 |
| 2.967096 | 4.199002 |
| 2.971968 | 4.215461 |
| 3.00737 | 4.219067 |
| 3.032521 | 4.25187 |
| 3.059956 | 4.288865 |
| 3.083682 | 4.317324 |
| 3.169026 | 4.343436 |
| 3.17159 | 4.432404 |
| 3.180263 | 4.435167 |
| 3.181319 | 4.444854 |
| 3.181873 | 4.445802 |
| 3.183838 | 4.446346 |
| 3.204309 | 4.448766 |
| 3.208173 | 4.473976 |
| 3.224944 | 4.478279 |
| 3.233772 | 4.497441 |
| 3.234281 | 4.504954 |
| 3.236396 | 4.505392 |
| 3.249477 | 4.507759 |
| 3.276336 | 4.523063 |
| 3.279042 | 4.554611 |
| 3.282128 | 4.557728 |
| 3.29121 | 4.561387 |
| 3.311443 | 4.572484 |
| 3.315431 | 4.597595 |
| 3.3189 | 4.602565 |
| 3.324838 | 4.606976 |
| 3.338964 | 4.614742 |
| 3.343294 | 4.633717 |
| 3.346551 | 4.639678 |
| 3.350006 | 4.644286 |
| 3.358781 | 4.649346 |
| 3.36249 | 4.662801 |
| 3.364938 | 4.668858 |
| 3.36653 | 4.6731 |
| 3.370785 | 4.676028 |
| 3.37289 | 4.684685 |
| 3.37393 | 4.689914 |
| 3.374268 | 4.693219 |
| 3.37488 | 4.694582 |
| 3.374382 | 4.699221 |
| 3.373414 | 4.702673 |
| 3.373104 | 4.704465 |
| 3.370979 | 4.704832 |
| 3.366866 | 4.706278 |
| 3.363295 | 4.706984 |
| 3.36294 | 4.706685 |
| 3.35901 | 4.706625 |
| 3.350264 | 4.70574 |
| 3.343498 | 4.702716 |
| 3.338916 | 4.699748 |
| 3.338138 | 4.697509 |
| 3.324514 | 4.697142 |
| 3.334827 | 4.689764 |
| 3.338284 | 4.703485 |
| 3.345105 | 4.708102 |
| 3.358242 | 4.716898 |
| 3.372037 | 4.734179 |
| 3.373469 | 4.75201 |
| 3.393891 | 4.753903 |
| 3.399528 | 4.780744 |
| 3.401666 | 4.787196 |
| 3.415545 | 4.789659 |
| 3.417169 | 4.807551 |
| 3.448393 | 4.809554 |
| 3.46274 | 4.848855 |
| 3.464527 | 4.866708 |
| 3.470232 | 4.869075 |
| 3.476936 | 4.876144 |
| 3.504852 | 4.884957 |
| 3.520414 | 4.919186 |
| 3.528504 | 4.938486 |
| 3.531228 | 4.948475 |
| 3.568914 | 4.951796 |
| 3.583208 | 4.998213 |
| 3.592906 | 5.015797 |
| 3.600152 | 5.027686 |
| 3.640582 | 5.036519 |
| 3.652905 | 5.085951 |
| 3.663663 | 5.101012 |
| 3.676994 | 5.114128 |
| 3.71985 | 5.130314 |
| 3.729494 | 5.182404 |
| 3.740272 | 5.194131 |
| 3.761736 | 5.207212 |
| 3.806705 | 5.233172 |
| 3.812961 | 5.287569 |
| 3.821932 | 5.295145 |
| 3.854352 | 5.305996 |
| 3.901128 | 5.345074 |
| 3.903283 | 5.401435 |
| 3.907292 | 5.404038 |
| 3.943943 | 5.408879 |
| 3.954809 | 5.45304 |
| 3.996424 | 5.465994 |
| 4.000412 | 5.515977 |
| 4.003093 | 5.520765 |
| 4.003093 | 5.523981 |

| Pressure | |
| --- | --- |
| Without nanoparticle | With nanoparticle |
| 1109.28 | 1451.17 |
| 1109.281 | 1451.169 |
| 1109.281 | 1451.169 |
| 1109.282 | 1451.168 |
| 1109.284 | 1451.163 |
| 1109.285 | 1451.162 |
| 1109.285 | 1451.161 |
| 1109.285 | 1451.159 |
| 1109.287 | 1451.153 |
| 1109.287 | 1451.151 |
| 1109.287 | 1451.15 |
| 1109.287 | 1451.147 |
| 1109.287 | 1451.14 |
| 1109.287 | 1451.139 |
| 1109.287 | 1451.138 |
| 1109.287 | 1451.132 |
| 1109.286 | 1451.125 |
| 1109.286 | 1451.124 |
| 1109.286 | 1451.123 |
| 1109.284 | 1451.115 |
| 1109.283 | 1451.107 |
| 1109.283 | 1451.107 |
| 1109.282 | 1451.106 |
| 1109.28 | 1451.095 |
| 1109.279 | 1451.093 |
| 1109.278 | 1451.087 |
| 1109.278 | 1451.087 |
| 1109.278 | 1451.087 |
| 1109.273 | 1451.072 |
| 1109.271 | 1451.065 |
| 1109.271 | 1451.065 |
| 1109.271 | 1451.064 |
| 1109.265 | 1451.047 |
| 1109.263 | 1451.041 |
| 1109.262 | 1451.04 |
| 1109.262 | 1451.038 |
| 1109.254 | 1451.018 |
| 1109.253 | 1451.014 |
| 1109.252 | 1451.013 |
| 1109.251 | 1451.01 |
| 1109.241 | 1450.987 |
| 1109.241 | 1450.985 |
| 1109.24 | 1450.984 |
| 1109.238 | 1450.979 |
| 1109.222 | 1450.95 |
| 1109.221 | 1450.95 |
| 1109.22 | 1450.947 |
| 1109.218 | 1450.944 |
| 1109.215 | 1450.938 |
| 1109.202 | 1450.915 |
| 1109.2 | 1450.913 |
| 1109.198 | 1450.908 |
| 1109.183 | 1450.883 |
| 1109.182 | 1450.881 |
| 1109.179 | 1450.876 |
| 1109.178 | 1450.874 |
| 1109.178 | 1450.873 |
| 1109.163 | 1450.847 |
| 1109.159 | 1450.839 |
| 1109.159 | 1450.839 |
| 1109.158 | 1450.838 |
| 1109.146 | 1450.816 |
| 1109.145 | 1450.814 |
| 1109.139 | 1450.804 |
| 1109.139 | 1450.803 |
| 1109.138 | 1450.803 |
| 1109.127 | 1450.782 |
| 1109.121 | 1450.771 |
| 1109.12 | 1450.769 |
| 1109.119 | 1450.767 |
| 1109.11 | 1450.75 |
| 1109.104 | 1450.739 |
| 1109.102 | 1450.735 |
| 1109.1 | 1450.733 |
| 1109.094 | 1450.72 |
| 1109.087 | 1450.708 |
| 1109.085 | 1450.703 |
| 1109.084 | 1450.701 |
| 1109.079 | 1450.691 |
| 1109.072 | 1450.678 |
| 1109.069 | 1450.672 |
| 1109.069 | 1450.671 |
| 1109.065 | 1450.663 |
| 1109.059 | 1450.65 |
| 1109.053 | 1450.636 |
| 1109.053 | 1450.636 |
| 1109.053 | 1450.635 |
| 1109.04 | 1450.607 |
| 1109.039 | 1450.606 |
| 1109.027 | 1450.579 |
| 1109.024 | 1450.574 |
| 1109.018 | 1450.561 |
| 1109.002 | 1450.527 |
| 1108.998 | 1450.521 |
| 1108.993 | 1450.514 |
| 1108.993 | 1450.513 |
| 1108.993 | 1450.512 |
| 1108.99 | 1450.509 |
| 1108.986 | 1450.504 |
| 1108.973 | 1450.481 |
| 1108.966 | 1450.466 |
| 1108.956 | 1450.45 |
| 1108.953 | 1450.444 |
| 1108.941 | 1450.423 |
| 1108.94 | 1450.421 |
| 1108.939 | 1450.418 |
| 1108.926 | 1450.398 |
| 1108.923 | 1450.392 |
| 1108.914 | 1450.377 |
| 1108.896 | 1450.352 |
| 1108.895 | 1450.348 |
| 1108.892 | 1450.341 |
| 1108.892 | 1450.34 |
| 1108.891 | 1450.338 |
| 1108.891 | 1450.337 |
| 1108.89 | 1450.333 |
| 1108.889 | 1450.332 |
| 1108.888 | 1450.327 |
| 1108.87 | 1450.301 |
| 1108.866 | 1450.294 |
| 1108.862 | 1450.286 |
| 1108.854 | 1450.274 |
| 1108.853 | 1450.269 |
| 1108.85 | 1450.261 |
| 1108.847 | 1450.257 |
| 1108.847 | 1450.256 |
| 1108.842 | 1450.24 |
| 1108.841 | 1450.238 |
| 1108.839 | 1450.231 |
| 1108.838 | 1450.222 |
| 1108.839 | 1450.206 |
| 1108.839 | 1450.205 |
| 1108.845 | 1450.199 |
| 1108.845 | 1450.199 |
| 1108.846 | 1450.207 |
| 1108.866 | 1450.208 |
| 1108.869 | 1450.213 |
| 1108.881 | 1450.215 |
| 1108.885 | 1450.229 |
| 1108.911 | 1450.238 |
| 1108.924 | 1450.241 |
| 1108.93 | 1450.244 |
| 1108.936 | 1450.263 |
| 1108.967 | 1450.279 |
| 1108.993 | 1450.282 |
| 1108.993 | 1450.283 |
| 1108.997 | 1450.296 |
| 1109.026 | 1450.306 |
| 1109.026 | 1450.331 |
| 1109.032 | 1450.332 |
| 1109.066 | 1450.336 |
| 1109.067 | 1450.356 |
| 1109.072 | 1450.385 |
| 1109.104 | 1450.394 |
| 1109.142 | 1450.399 |
| 1109.151 | 1450.41 |
| 1109.16 | 1450.443 |
| 1109.182 | 1450.466 |
| 1109.223 | 1450.467 |
| 1109.247 | 1450.469 |
| 1109.255 | 1450.478 |
| 1109.264 | 1450.495 |
| 1109.307 | 1450.506 |
| 1109.322 | 1450.527 |
| 1109.349 | 1450.537 |
| 1109.353 | 1450.547 |
| 1109.381 | 1450.572 |
| 1109.402 | 1450.584 |
| 1109.434 | 1450.596 |
| 1109.477 | 1450.621 |
| 1109.477 | 1450.635 |
| 1109.478 | 1450.64 |
| 1109.494 | 1450.652 |
| 1109.528 | 1450.666 |
| 1109.537 | 1450.667 |
| 1109.538 | 1450.674 |
| 1109.567 | 1450.682 |
| 1109.577 | 1450.685 |
| 1109.589 | 1450.704 |
| 1109.6 | 1450.711 |
| 1109.64 | 1450.715 |
| 1109.659 | 1450.723 |
| 1109.661 | 1450.73 |
| 1109.675 | 1450.754 |
| 1109.714 | 1450.775 |
| 1109.737 | 1450.784 |
| 1109.745 | 1450.792 |
| 1109.753 | 1450.805 |
| 1109.766 | 1450.86 |
| 1109.839 | 1450.863 |
| 1109.843 | 1450.877 |
| 1109.853 | 1450.901 |
| 1109.891 | 1450.905 |
| 1109.901 | 1450.94 |
| 1109.941 | 1450.96 |
| 1109.982 | 1451.011 |
| 1110.029 | 1451.039 |
| 1110.067 | 1451.049 |
| 1110.082 | 1451.072 |
| 1110.12 | 1451.131 |
| 1110.175 | 1451.164 |
| 1110.184 | 1451.183 |
| 1110.241 | 1451.188 |
| 1110.262 | 1451.208 |
| 1110.322 | 1451.225 |
| 1110.348 | 1451.253 |
| 1110.401 | 1451.301 |
| 1110.414 | 1451.304 |
| 1110.424 | 1451.326 |
| 1110.465 | 1451.327 |
| 1110.482 | 1451.37 |
| 1110.534 | 1451.413 |
| 1110.571 | 1451.45 |
| 1110.597 | 1451.475 |
| 1110.619 | 1451.499 |
| 1110.658 | 1451.524 |
| 1110.667 | 1451.529 |
| 1110.691 | 1451.543 |
| 1110.717 | 1451.557 |
| 1110.732 | 1451.563 |
| 1110.76 | 1451.591 |
| 1110.776 | 1451.603 |
| 1110.778 | 1451.604 |
| 1110.78 | 1451.604 |
| 1110.796 | 1451.606 |
| 1110.802 | 1451.609 |
| 1110.818 | 1451.618 |
| 1110.834 | 1451.629 |
| 1110.863 | 1451.645 |
| 1110.863 | 1451.646 |
| 1110.871 | 1451.649 |
| 1110.873 | 1451.649 |
| 1110.875 | 1451.651 |
| 1110.883 | 1451.652 |
| 1110.884 | 1451.653 |
| 1110.886 | 1451.655 |
| 1110.9 | 1451.652 |
| 1110.902 | 1451.65 |
| 1110.905 | 1451.644 |
| 1110.906 | 1451.634 |
| 1110.906 | 1451.63 |
| 1110.903 | 1451.617 |
| 1110.891 | 1451.585 |
| 1110.891 | 1451.585 |
| 1110.89 | 1451.585 |
| 1110.876 | 1451.585 |
| 1110.874 | 1451.554 |
| 1110.864 | 1451.55 |
| 1110.858 | 1451.529 |
| 1110.856 | 1451.52 |
| 1110.841 | 1451.518 |
| 1110.829 | 1451.498 |
| 1110.815 | 1451.473 |
| 1110.8 | 1451.451 |
| 1110.736 | 1451.429 |
| 1110.733 | 1451.334 |
| 1110.725 | 1451.33 |
| 1110.724 | 1451.318 |
| 1110.724 | 1451.316 |
| 1110.723 | 1451.316 |
| 1110.711 | 1451.314 |
| 1110.708 | 1451.295 |
| 1110.698 | 1451.291 |
| 1110.692 | 1451.275 |
| 1110.692 | 1451.269 |
| 1110.69 | 1451.269 |
| 1110.681 | 1451.267 |
| 1110.661 | 1451.253 |
| 1110.659 | 1451.224 |
| 1110.656 | 1451.221 |
| 1110.649 | 1451.217 |
| 1110.63 | 1451.205 |
| 1110.626 | 1451.178 |
| 1110.622 | 1451.172 |
| 1110.615 | 1451.167 |
| 1110.598 | 1451.157 |
| 1110.592 | 1451.132 |
| 1110.587 | 1451.123 |
| 1110.582 | 1451.116 |
| 1110.566 | 1451.108 |
| 1110.558 | 1451.085 |
| 1110.552 | 1451.074 |
| 1110.547 | 1451.065 |
| 1110.533 | 1451.059 |
| 1110.523 | 1451.039 |
| 1110.515 | 1451.025 |
| 1110.512 | 1451.014 |
| 1110.5 | 1451.01 |
| 1110.487 | 1450.992 |
| 1110.479 | 1450.975 |
| 1110.477 | 1450.963 |
| 1110.466 | 1450.96 |
| 1110.451 | 1450.945 |
| 1110.441 | 1450.925 |
| 1110.441 | 1450.912 |
| 1110.431 | 1450.91 |
| 1110.414 | 1450.898 |
| 1110.403 | 1450.875 |
| 1110.396 | 1450.86 |
| 1110.395 | 1450.851 |
| 1110.377 | 1450.849 |
| 1110.347 | 1450.825 |
| 1110.337 | 1450.789 |
| 1110.319 | 1450.777 |
| 1110.287 | 1450.756 |
| 1110.256 | 1450.716 |
| 1110.253 | 1450.679 |
| 1110.212 | 1450.675 |
| 1110.201 | 1450.623 |
| 1110.197 | 1450.612 |
| 1110.172 | 1450.607 |
| 1110.169 | 1450.576 |
| 1110.119 | 1450.573 |
| 1110.098 | 1450.511 |
| 1110.095 | 1450.486 |
| 1110.087 | 1450.482 |
| 1110.078 | 1450.472 |
| 1110.042 | 1450.46 |
| 1110.024 | 1450.416 |
| 1110.014 | 1450.393 |
| 1110.011 | 1450.382 |
| 1109.971 | 1450.378 |
| 1109.957 | 1450.328 |
| 1109.948 | 1450.31 |
| 1109.942 | 1450.299 |
| 1109.907 | 1450.29 |
| 1109.897 | 1450.246 |
| 1109.889 | 1450.234 |
| 1109.879 | 1450.223 |
| 1109.85 | 1450.211 |
| 1109.844 | 1450.173 |
| 1109.838 | 1450.165 |
| 1109.825 | 1450.156 |
| 1109.802 | 1450.14 |
| 1109.799 | 1450.108 |
| 1109.795 | 1450.104 |
| 1109.781 | 1450.098 |
| 1109.763 | 1450.079 |
| 1109.762 | 1450.054 |
| 1109.761 | 1450.053 |
| 1109.749 | 1450.051 |
| 1109.746 | 1450.034 |
| 1109.736 | 1450.029 |
| 1109.735 | 1450.013 |
| 1109.734 | 1450.012 |
| 1109.734 | 1450.011 |

| Velocity | |
| --- | --- |
| Without nanoparticle | With nanoparticle |
| 0.027277 | 0.026181 |
| 0.028788 | 0.027632 |
| 0.029594 | 0.028406 |
| 0.030915 | 0.029675 |
| 0.036427 | 0.034967 |
| 0.037664 | 0.036155 |
| 0.038527 | 0.036984 |
| 0.040402 | 0.038784 |
| 0.045434 | 0.043614 |
| 0.046401 | 0.044542 |
| 0.047271 | 0.045377 |
| 0.049724 | 0.047731 |
| 0.054296 | 0.05212 |
| 0.054996 | 0.052791 |
| 0.055805 | 0.053567 |
| 0.058881 | 0.056519 |
| 0.063016 | 0.060485 |
| 0.063453 | 0.060903 |
| 0.064104 | 0.061528 |
| 0.06788 | 0.065149 |
| 0.071599 | 0.068714 |
| 0.071776 | 0.068884 |
| 0.072122 | 0.069215 |
| 0.076727 | 0.073628 |
| 0.077444 | 0.074314 |
| 0.079896 | 0.076662 |
| 0.079974 | 0.076737 |
| 0.080051 | 0.076811 |
| 0.085433 | 0.081964 |
| 0.087796 | 0.084224 |
| 0.088054 | 0.084471 |
| 0.088381 | 0.084783 |
| 0.094005 | 0.090162 |
| 0.09567 | 0.091752 |
| 0.096022 | 0.092088 |
| 0.096595 | 0.092636 |
| 0.102451 | 0.098228 |
| 0.1035 | 0.099229 |
| 0.103884 | 0.099595 |
| 0.104702 | 0.100375 |
| 0.110782 | 0.10617 |
| 0.11128 | 0.106644 |
| 0.111649 | 0.106996 |
| 0.112712 | 0.108008 |
| 0.120598 | 0.115485 |
| 0.120861 | 0.115734 |
| 0.12165 | 0.116482 |
| 0.122453 | 0.117241 |
| 0.123916 | 0.118622 |
| 0.130229 | 0.124586 |
| 0.13078 | 0.125106 |
| 0.13211 | 0.126359 |
| 0.138982 | 0.132831 |
| 0.139501 | 0.133319 |
| 0.140846 | 0.134584 |
| 0.141432 | 0.135135 |
| 0.141667 | 0.135355 |
| 0.148667 | 0.141929 |
| 0.150773 | 0.143904 |
| 0.150782 | 0.143912 |
| 0.151112 | 0.144222 |
| 0.157129 | 0.149854 |
| 0.157715 | 0.15041 |
| 0.160432 | 0.152953 |
| 0.160575 | 0.153087 |
| 0.160765 | 0.153266 |
| 0.166635 | 0.158756 |
| 0.169612 | 0.16154 |
| 0.170216 | 0.162105 |
| 0.170762 | 0.162616 |
| 0.175411 | 0.166961 |
| 0.178639 | 0.169976 |
| 0.179679 | 0.170949 |
| 0.18032 | 0.171547 |
| 0.184033 | 0.175017 |
| 0.1875 | 0.178256 |
| 0.188951 | 0.179612 |
| 0.189535 | 0.180159 |
| 0.192488 | 0.182919 |
| 0.196182 | 0.186372 |
| 0.198015 | 0.188087 |
| 0.198455 | 0.188499 |
| 0.200765 | 0.19066 |
| 0.204672 | 0.194319 |
| 0.209899 | 0.199243 |
| 0.20991 | 0.199251 |
| 0.209953 | 0.199583 |
| 0.219782 | 0.208552 |
| 0.220109 | 0.208768 |
| 0.228665 | 0.217033 |
| 0.230582 | 0.21863 |
| 0.234267 | 0.222317 |
| 0.243374 | 0.231692 |
| 0.245158 | 0.233073 |
| 0.247448 | 0.234829 |
| 0.24747 | 0.235069 |
| 0.247493 | 0.23532 |
| 0.248629 | 0.236009 |
| 0.250581 | 0.237166 |
| 0.255927 | 0.24275 |
| 0.25892 | 0.246158 |
| 0.263201 | 0.250091 |
| 0.264308 | 0.251283 |
| 0.26957 | 0.256217 |
| 0.2699 | 0.256851 |
| 0.27024 | 0.257511 |
| 0.275413 | 0.262152 |
| 0.276996 | 0.263519 |
| 0.280409 | 0.267138 |
| 0.288211 | 0.273314 |
| 0.288843 | 0.274354 |
| 0.289863 | 0.276042 |
| 0.290011 | 0.276267 |
| 0.290246 | 0.276634 |
| 0.290447 | 0.277005 |
| 0.291046 | 0.278002 |
| 0.291218 | 0.278306 |
| 0.291964 | 0.27962 |
| 0.30001 | 0.286439 |
| 0.302244 | 0.288306 |
| 0.30421 | 0.29052 |
| 0.308314 | 0.294207 |
| 0.309674 | 0.296166 |
| 0.312339 | 0.299501 |
| 0.31571 | 0.301409 |
| 0.31602 | 0.301593 |
| 0.324156 | 0.309491 |
| 0.325137 | 0.310371 |
| 0.327836 | 0.312729 |
| 0.331416 | 0.31635 |
| 0.339491 | 0.324341 |
| 0.340176 | 0.325065 |
| 0.34528 | 0.329941 |
| 0.345537 | 0.330158 |
| 0.345855 | 0.336483 |
| 0.351907 | 0.337702 |
| 0.352845 | 0.340196 |
| 0.355748 | 0.341303 |
| 0.356748 | 0.346671 |
| 0.362298 | 0.349345 |
| 0.364667 | 0.350112 |
| 0.36571 | 0.351095 |
| 0.366681 | 0.355598 |
| 0.371466 | 0.358754 |
| 0.374859 | 0.35928 |
| 0.374877 | 0.359604 |
| 0.37539 | 0.361746 |
| 0.378828 | 0.36333 |
| 0.378847 | 0.366902 |
| 0.379464 | 0.367067 |
| 0.382927 | 0.367613 |
| 0.38299 | 0.369931 |
| 0.383502 | 0.373057 |
| 0.386342 | 0.373915 |
| 0.389345 | 0.374452 |
| 0.390023 | 0.375469 |
| 0.390675 | 0.378141 |
| 0.392151 | 0.379798 |
| 0.394696 | 0.379902 |
| 0.396053 | 0.380009 |
| 0.396457 | 0.380618 |
| 0.396941 | 0.381775 |
| 0.399032 | 0.382449 |
| 0.399687 | 0.383662 |
| 0.400782 | 0.384206 |
| 0.400972 | 0.384742 |
| 0.401986 | 0.385944 |
| 0.402711 | 0.386445 |
| 0.403717 | 0.386887 |
| 0.404935 | 0.387789 |
| 0.404949 | 0.38827 |
| 0.404955 | 0.38847 |
| 0.405412 | 0.388955 |
| 0.406294 | 0.389508 |
| 0.406518 | 0.38953 |
| 0.406547 | 0.390078 |
| 0.406986 | 0.390307 |
| 0.407065 | 0.390379 |
| 0.407132 | 0.390574 |
| 0.407173 | 0.3906 |
| 0.407165 | 0.390606 |
| 0.407097 | 0.390606 |
| 0.407086 | 0.390594 |
| 0.407014 | 0.390472 |
| 0.406736 | 0.390288 |
| 0.406525 | 0.390185 |
| 0.406439 | 0.39009 |
| 0.406357 | 0.389925 |
| 0.406207 | 0.389009 |
| 0.405168 | 0.388934 |
| 0.405097 | 0.388669 |
| 0.40493 | 0.388146 |
| 0.404236 | 0.388037 |
| 0.404039 | 0.387191 |
| 0.403194 | 0.386629 |
| 0.402236 | 0.385106 |
| 0.401029 | 0.384185 |
| 0.399963 | 0.383844 |
| 0.399518 | 0.383018 |
| 0.398312 | 0.380655 |
| 0.396441 | 0.37916 |
| 0.396093 | 0.378277 |
| 0.393885 | 0.378042 |
| 0.39303 | 0.377057 |
| 0.390325 | 0.376153 |
| 0.389067 | 0.374644 |
| 0.38626 | 0.371715 |
| 0.38556 | 0.371571 |
| 0.384985 | 0.370107 |
| 0.382535 | 0.37006 |
| 0.38141 | 0.366922 |
| 0.377873 | 0.363472 |
| 0.375555 | 0.360921 |
| 0.373339 | 0.358291 |
| 0.371288 | 0.355427 |
| 0.367172 | 0.35189 |
| 0.366057 | 0.351056 |
| 0.363113 | 0.348818 |
| 0.359361 | 0.346278 |
| 0.356976 | 0.344892 |
| 0.352035 | 0.338065 |
| 0.348663 | 0.334008 |
| 0.347957 | 0.333717 |
| 0.34717 | 0.333391 |
| 0.340399 | 0.325651 |
| 0.339068 | 0.324755 |
| 0.33507 | 0.321324 |
| 0.330605 | 0.316705 |
| 0.320908 | 0.307086 |
| 0.320731 | 0.306773 |
| 0.317643 | 0.303663 |
| 0.316919 | 0.303539 |
| 0.315683 | 0.3022 |
| 0.312282 | 0.299858 |
| 0.311723 | 0.298664 |
| 0.310408 | 0.296017 |
| 0.298348 | 0.285468 |
| 0.295714 | 0.283136 |
| 0.290202 | 0.276996 |
| 0.282305 | 0.269514 |
| 0.279103 | 0.2668 |
| 0.272021 | 0.26012 |
| 0.259113 | 0.24706 |
| 0.258666 | 0.247053 |
| 0.25785 | 0.247042 |
| 0.247853 | 0.247036 |
| 0.246662 | 0.23684 |
| 0.241514 | 0.235589 |
| 0.238096 | 0.229449 |
| 0.237201 | 0.226938 |
| 0.230511 | 0.226379 |
| 0.22555 | 0.221158 |
| 0.219925 | 0.214934 |
| 0.214866 | 0.209874 |
| 0.194943 | 0.204996 |
| 0.194297 | 0.186252 |
| 0.192088 | 0.185605 |
| 0.191817 | 0.1833 |
| 0.191725 | 0.183072 |
| 0.191397 | 0.182995 |
| 0.187867 | 0.182655 |
| 0.187175 | 0.179001 |
| 0.184061 | 0.178356 |
| 0.182345 | 0.1754 |
| 0.182245 | 0.174201 |
| 0.181824 | 0.174131 |
| 0.179142 | 0.173748 |
| 0.173117 | 0.171209 |
| 0.172464 | 0.165591 |
| 0.171707 | 0.165004 |
| 0.169398 | 0.164306 |
| 0.163731 | 0.162132 |
| 0.162507 | 0.156835 |
| 0.161408 | 0.155714 |
| 0.159442 | 0.154695 |
| 0.154217 | 0.152844 |
| 0.152411 | 0.147951 |
| 0.150968 | 0.146282 |
| 0.149342 | 0.144939 |
| 0.144605 | 0.143406 |
| 0.14221 | 0.138959 |
| 0.140425 | 0.136733 |
| 0.139142 | 0.135065 |
| 0.134925 | 0.133852 |
| 0.131942 | 0.12988 |
| 0.12982 | 0.127092 |
| 0.128884 | 0.125101 |
| 0.125211 | 0.12421 |
| 0.121644 | 0.120737 |
| 0.119196 | 0.117385 |
| 0.118605 | 0.115075 |
| 0.115493 | 0.114509 |
| 0.111358 | 0.111551 |
| 0.108597 | 0.10764 |
| 0.108347 | 0.105019 |
| 0.105807 | 0.104776 |
| 0.101121 | 0.102347 |
| 0.098065 | 0.097886 |
| 0.096185 | 0.094963 |
| 0.095878 | 0.093149 |
| 0.090976 | 0.092873 |
| 0.088138 | 0.088151 |
| 0.087231 | 0.085402 |
| 0.085497 | 0.084513 |
| 0.082338 | 0.082866 |
| 0.079231 | 0.07978 |
| 0.07892 | 0.076774 |
| 0.074656 | 0.076465 |
| 0.073532 | 0.072236 |
| 0.073112 | 0.07126 |
| 0.070448 | 0.070892 |
| 0.070143 | 0.068274 |
| 0.064534 | 0.067987 |
| 0.062093 | 0.06257 |
| 0.061794 | 0.060228 |
| 0.060848 | 0.059923 |
| 0.059749 | 0.059017 |
| 0.055319 | 0.0579 |
| 0.052939 | 0.05369 |
| 0.051725 | 0.051395 |
| 0.05132 | 0.050228 |
| 0.045864 | 0.049843 |
| 0.043863 | 0.044598 |
| 0.042525 | 0.042673 |
| 0.041534 | 0.041389 |
| 0.036146 | 0.040443 |
| 0.034546 | 0.035277 |
| 0.033164 | 0.033741 |
| 0.03147 | 0.032417 |
| 0.026148 | 0.0308 |
| 0.024975 | 0.02571 |
| 0.023674 | 0.024586 |
| 0.021114 | 0.023342 |
| 0.015873 | 0.0209 |
| 0.015157 | 0.015895 |
| 0.014137 | 0.01521 |
| 0.01051 | 0.014234 |
| 0.005536 | 0.010769 |
| 0.005322 | 0.005966 |
| 0.004928 | 0.005753 |
| 0.002375 | 0.005362 |
| 0.002506 | 0.002357 |
| 0.005828 | 0.002068 |
| 0.006219 | 0.004695 |
| 0.006484 | 0.00506 |
| 0.006484 | 0.005307 |
